# Assessment of Transcatheter or Surgical Closure of Atrial Septal Defect using Interpretable Deep Keypoint Stadiometry

**DOI:** 10.34133/2022/9790653

**Published:** 2022-10-21

**Authors:** Jing Wang, Wanqing Xie, Mingmei Cheng, Qun Wu, Fangyun Wang, Pei Li, Bo Fan, Xin Zhang, Binbin Wang, Xiaofeng Liu

**Affiliations:** ^1^School of Basic Medical Sciences, Capital Medical University, Beijing 10069, China; ^2^Department of Intelligent Medical Engineering, School of Biomedical Engineering, Anhui Medical University, Hefei 230032, China; ^3^Beth Israel Deaconess Medical Center, Harvard Medical School, Harvard University, Boston, MA 02215, USA; ^4^Heart Center, Beijing Children's Hospital, Capital Medical University, National Center for Children's Health, Beijing 10045, China; ^5^Center for Genetics, National Research Institute for Family Planning, Beijing 100730, China; ^6^Graduated school, Peking Union Medical College, Beijing 100730, China; ^7^Gordon Center for Medical Imaging, Harvard Medical School, and Massachusetts General Hospital, Boston, MA 02114, USA

## Abstract

Automated echocardiogram interpretation with artificial intelligence (AI) has the potential to facilitate the serial diagnosis of heart defects by primary clinician. However, the fully automated and interpretable analysis pipeline for suggesting a treatment plan is largely underexplored. The present study targets to build an automatic and interpretable assistant for the transthoracic echocardiogram- (TTE-) based assessment of atrial septal defect (ASD) with deep learning (DL). We developed a novel deep keypoint stadiometry (DKS) model, which learns to precisely localize the keypoints, i.e., the endpoints of defects and followed by the absolute distance measurement with the scale. The closure plan and the size of the ASD occluder for transcatheter closure are derived based on the explicit clinical decision rules. A total of 3,474 2D and Doppler TTE from 579 patients were retrospectively collected from two clinical groups. The accuracy of closure classification using DKS (0.9425 ± 0.0052) outperforms the “black-box” model (0.7646 ± 0.0068; *p* < 0.0001) for within-center evaluation. The results in cross-center cases or using the quadratic weighted kappa as an evaluation metric are consistent. The fine-grained keypoint label provides more explicit supervision for network training. While DKS can be fully automated, clinicians can intervene and edit at different steps of the process as well. Our deep learning keypoint localization can provide an automatic and transparent way for assessing size-sensitive congenital heart defects, which has huge potential value for application in primary medical institutions in China. Also, more size-sensitive treatment planning tasks may be explored in the future.

## 1. Introduction

Atrial septal defect (ASD) is a typical cardiac defect with a hole in the atrial septum, which accounts for around 10% of congenital heart disease (CHD) and about 0.1% of live births [1, 2] . ASD can be the cause of congestive heart failure or stroke. Thus, the early diagnosis and treatment of ASD are crucial to avoid serious complications. Surgical closure has been the gold standard solution for ASD for a few decades [3, 4] . In recent years, noninvasive/minimally invasive closure treatment has been commonly recommended [5]. Targeting the minimally invasive ASD closure, the occluder can be inserted into the heart using a catheter from the groin [6]. Since the first attempt at transcatheter closure in 1976 [7], it has become more common as a primary therapeutic solution [8, 9] . However, the transcatheter closure with occluder requires a limited size of the ASD and sufficient rim all around [10–12]. Therefore, suggesting an appropriate therapeutic plan, i.e., transcatheter or surgical, is an important step in clinical practice [13].

Conventional pretreatment assessment/intraoperative guidance methods to measure critical scales of ASD include 2D transthoracic echocardiogram (TTE), 2D transesophageal echocardiogram (TEE), and 3D TEE. In adult ASD assessment, the assessment approach could be accomplished through TEE guidance [14]. But for children with relatively thinner chest and abdomen walls, while too young to tolerate TEE, 2D TTE is an excellent approach for pretreatment assessment and intraoperative guidance [15], which is commonly used in most primary hospitals in China.

As reported, the current Chinese pediatric health care system is facing huge challenges mainly including generally unmet demand and uneven development. There are only 4 pediatricians per 10 thousand children and large number of inadequately trained pediatricians who work in pediatrics of primary hospitals [16]. Thus, although 2D TTE is reliable and mostly used, it has a potential risk of measurement bias due to the subjective judgment by inadequately trained pediatricians. Due to the irregular shape of ASD and the restricted windows of the machine, the measurement of ASD size/location and rims could be inaccurate, leading to deviations in the assessment of transcatheter or surgical closure, resulting in unnecessary surgery or complications like device embolization or device erosion [17, 18].

Artificial intelligence (AI), especially the recent deep learning (DL), has been widely applied in medical image analysis and is on par with the human expert performance in many tasks [19]. Several studies have focused on the automated pipeline for cardiology disease detection, which has the potential to improve primary care in developing areas [20, 21]. In our previous study, a deep learning system showed superior performance in diagnosing the health and CHD samples with multiview TTE [22]. However, the AI for the subsequent ASD treatment planning has been largely neglected.

**Figure 1:**
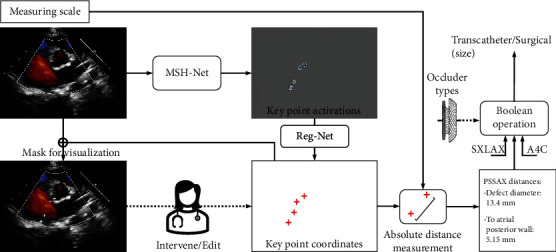
Illustration of our proposed processing flow with PSSAX of the aorta view. We use deep learning-based MSHNet and RegNet to precisely estimate the coordinates of the keypoints in each view. The clinicians can intervene or edit the keypoint coordinates by checking the masked visualizations. Then, the absolute distance is calculated based on the coordinates and the corresponding measuring scale. Boolean expressions based on the parameters of the occluder are applied to make the final decision.

To our knowledge, the current study is the first to suggest the ASD closure treatment plan automatically. A straightforward solution is to use a “black-box” binary classification model. However, the clinicians should still make the final decision in a real-world setting. We expect the AI to provide a more transparent and reliable computer-aided suggestion. In this context, we developed a novel deep learning-based AI model (deep keypoint stadiometry, DKS) to localize the endpoints of defects, i.e., keypoint of CHD, in multiview TTE accurately and therefore differentiate the transcatheter or surgical closure following the explicit expert knowledge-based clinical rules. Notably, the novel multiscale hourglass network (MSHNet) and anatomical-aware supervision (AAS) loss are proposed to improve the performance in medical image keypoint localization. By automating each step in real-world clinical practice, our system can derive more interpretable suggestions and allow manual correction of some of the intermediate keypoint localization results in a cooperative manner. The processing flow of our system is shown in Figure 1. The clinical team can intervene and edit or supersede AI with clinical acumen when deemed necessary.

We investigated the performance of the proposed “black-box” and DKS models in different TTE views with respect to the closure plan classification and occluder size prediction in a dataset with 2,700 2D and Doppler TTE from 450 participants. According to the results, the accuracy of transcatheter or surgical closure classification using DKS (0.9425 ± 0.0052) was significantly higher than that of the “black-box” model (0.7646 ± 0.0068; *p* < 0.0001). In addition, the Doppler TTE (0.9425 ± 0.0052) outperforms the 2D-TTE (0.8781 ± 0.0053; *p* < 0.0001) in keypoint localization and the subsequent closure classification. The generality of our model is further evidenced in a second evaluation dataset with 774 TTE from 129 patients. The proposed DKS model has high keypoint localization performance in multiview TTE. With the evidence-based clinical decision rules, surgeons could efficiently use this algorithm to conclude an interpretable operative plan. The proposed DKS model can be potentially applied to alleviate the keypoint labeling and measuring work of the preliminary clinicians. While it can be fully automated, experienced clinicians can intervene and edit at different steps of the process as well. Similar studies using deep learning algorithms shall be developed for size-sensitive treatments in the future.

## 2. Results

To test whether the proposed “black-box” model and DKS model can assist in therapy suggestions and verify whether the DSK model has good interpretability, especially whether it can provide clinicians with more details through positioning keypoints of the defect area, we conducted different experiments. In the present study, a total of 5 different models were evaluated. The two basic models include the “black-box” model and our proposed DKS model. In addition, three DKS derivative models were used for ablation studies. The “DKS w/o MSH” indicates using single-scale hourglass networks rather than MSHNet. The “DKS w/o AAS” denotes without using anatomical-aware supervision. “DKS w/o MSH&AAS” can be the vanilla SHN [23].

### 2.1. Keypoint Localization

**Figure 2:**
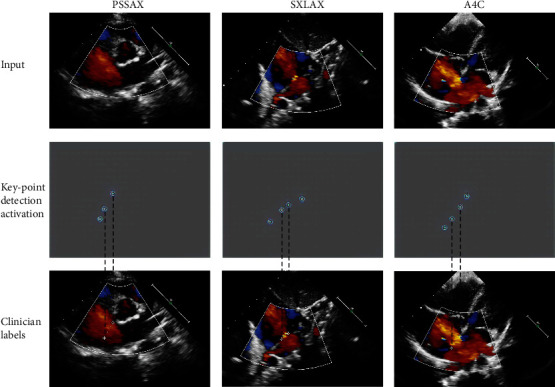
The keypoint activation in each view and its corresponding clinicians' labels for measuring the defect diameter (white cross). PSSAX: short for the aorta PSSAX of the aorta, SXLAX is short for SXLAX of two atria.

Correct inference of the positions of keypoints is very important to measure the size of the defect area, thus affecting the final treatment suggestions. To measure the accuracy of keypoint location, we conducted different experiments, and the PCK results of each view from the first dataset are shown in Table 1(a). According to the results, the DKS using Doppler TTE (0.9858 ± 0.0034) outperforms 2D TTE (0.8938 ± 0.0041) by 9.2%. In the ablation study, the proposed DKS model achieved the best performance w.r.t. PCK over the DKS w/o MSH/AAS/MSH&AAS models. The performance on the second evaluation dataset is reported in Table 2(b), showing the same tendency as in the first dataset. The consistent performance demonstrated that our system could be robust. In Figure 2, we visualized the keypoint activation and compared it with the clinicians' labels, which are well aligned. Quantitative results show that our DKS model can accurately predict the keypoints (including the cross-center evaluation set). The visual results also prove the reliability of our DKS model and further show the good interpretability of the DKS model-based treatment scheme classifier.

### 2.2. Binary-Therapy Classification

We adopt various experiments with different models to conduct the transcatheter and surgical closure classification. The comparisons of classification performance are shown in Table 2(a). The classification performance of DKS using Doppler TTE exhibits significant improvements (*p* < 0.0001) compared with the DKS using 2D TTE and the three ablation designs of DKS: the “DKS w/o MSH” model, the “DKS w/o AAS” model, and the “DKS w/o MSH&AAS” model. We also compared the accuracy of the “black-box” model and the DKS model in Figure S1. The “black-box” model was saturated in relatively low accuracy. Similarly, we also evaluated on the other dataset and provided the results in Table 2(b). The experimental results show that compared with the “black-box” model, our DKS model is significantly better in classification accuracy and interpretability and reaches the ACC of 0.9192 on the cross-center evaluation set.

### 2.3. Occluder Size Prediction

In Figure S2, we compared the performance of ASD occluder size suggestions for the transcatheter closure cases. The MAE and QWK of our DKS model are 0.7341 ± 0.08752 and 0.9027 ± 0.01254, respectively. In addition, a failure case is analyzed in Supplementary Section 6.

## 3. Discussion

Multiview TTE plays a vital role in assessing and guiding ASD clinical treatments and procedures, which is critical for ASD prognosis. It could help us to determine the presence, location, size, and hemodynamic characteristics of ASD, which are essential for clinical assessment.

With the recent progress of deep learning [24], numerous convolutional neural network (CNN) models demonstrating high accuracy in many medical image analysis tasks have been developed [22, 25]. While deep learning has achieved impressive results for classification, many CNN models are seen as a “black-box” model [24].

The present study has investigated two possible solutions for suggesting transcatheter or surgical closure with multiview TTE. The “black-box” model is firstly proposed, which simply formulated the task as a binary classification problem and used a typical convolutional neural network to learn a mapping from the multiview TTE to the class label. However, it can be challenging to explore the causal relationship, and the processing is not transparent for monitoring. CNN models remain largely elusive, how a particular CNN model plans and if it can be transparently checked. Therefore, it is of great importance to develop a transparent model that can be reliably and efficiently applied for clinical applications. Moreover, we need a system that can be flexibly adapted to other kinds of occluder types, because the decision rules in the Boolean expressions can be adjusted based on the available occluder types. In contrast, the label used for “black-box” training is exclusive to a specific occluder type. In this case, the manual label of the dataset is required from the experienced clinicians for the new types of occluders, which can be costly or prohibitive. Instead, the keypoint localization model is general for occluder selection, and the subsequent Boolean operation can be flexibly changed accruing to the clinical guidance of the occluders.

Based on these concerns, we proposed a novel DKS model to imitate and automate real-world clinical practice. The DKS pipeline consists of three modules, i.e., keypoint localization, length measuring with scale, and decision with Boolean expressions. We strictly follow the clinical rules to derive the treatment plan and the appropriate occluder size for transcatheter closure, which can be more transparent than the “black-box” model.

The modular design makes the system more transparent and can be easily monitored. For example, the keypoint localization is visible to the clinicians. The system can provide either fully automated suggestions or computer-aided processing with intermediate localization results. While clinicians can intervene and edit at different steps of the process, work is being done for this to be fully automated as well. Based on the experience of the clinicians, it can be easy to find out the mismatch and manually correct the point locations. Therefore, we provided an additional monitoring and interaction window.

The DKS framework developed in this study has high PCK for keypoint localization, highly improved from the vanilla version of SHN [25] as evidenced in our ablation study. The application of the vanilla SHN can be hindered by several problems. Firstly, its scaling is unstable. A minor change of the input scale can lead to a large shift in the point location. However, the size of the heart can vary largely in real-world imaging, since the distance of the ultrasonic probe to the heart can be affected by the body size and pose. With a novel multiscale supervision, we could achieve a more stable performance for scale change. Secondly, the anatomical structure prior is not able to be incorporated. In this work, we propose to explicitly incorporate the anatomical structure prior for keypoint localization. In our ablation study, we have demonstrated that such prior could provide important cues in real-world scenarios and improve the performance by a large margin.

With accurate keypoint localization, we could achieve good transcatheter or surgical closure classification performance. The keypoint label provides fine-grained supervision over the class-level transcatheter or surgical label, which inherently introduces richer information in the network training. The explicit clinical practice rules can largely alleviate the difficulty of exploring the inherent causal relationships with deep learning. The keypoint localization is also promising in size-sensitive treatments and automated robotic surgery for ASD closure [26, 27] .

Thus, our automatic ASD closure treatment plan system has two possible clinical application scenarios. For primary clinicians/novice readers, either our “black-box” or “white-box” models can be used to provide timely treatment planning, while the DKS-based “white-box” model is able to provide higher accuracy. The keypoint is a more fine-grained label compared with the image-wise class label. In addition, the conditional statements used in our subsequent Boolean expressions strictly follow the clinical decision rules, which do not rely on a network to learn it. Therefore, superior performance of the DKS model over the “black-box” model can be expected. For experienced clinicians, our DKS model is able to speed up/partially automate the treatment planning in a computer-aided fashion. Currently, without the deep learning system, the clinicians do plot the coordinates of keypoints from the start and measure the absolute distance to decide the defect size. Our DKS model explicitly mimics this process flow and makes it automatic. However, this can be time-consuming for multiple views and requires untrivial clinical training to make accurate keypoint labeling. The experienced clinicians only need to adjust the keypoints in the checking stage when it is not correct.

The frequency of the intervention is related to the experience of the clinicians. For primary clinicians/novice readers, we do not expect the clinicians to precisely adjust the coordinates. Instead, in our first and second evaluation dataset, about 12% and 15% of keypoints can be manually adjusted by experienced clinicians to achieve 100% accuracy, respectively. We note that the reported results are based on the end-to-end DL models without manual interpretations.

Furthermore, in the medical system of China, the sonographers are engaged in diagnosis with medical images, while cardiologists perform further clinical operations. Thus, the ASD assessment data and treatment suggestions predicted by the current model will be given to the cardiologists before operation. Though some of the performance of this model needs to be readjusted by the cardiologist but will not lead to wrong treatment in clinical practice finally.

There are some limitations to this study. First, the present work only focuses on the closure planning for ASD. It is promising to include more echocardiograms of patients with other CHD subtypes in the following study to extend this diagnostic system to more CHD subtypes in the future. Second, the CHD diagnosis system [22] can be concatenated to form an end-to-end pipeline for diagnosis and treatment suggestions. Third, it is promising to extend our DKS to the adult population. We note that the closure plan decision for adults usually relies on both 2D and 3D TEE or ICE measurements along with balloon sizing. It can be helpful to develop a unified system to take all these measurements into consideration.

In conclusion, a transparent AI-based multiview echocardiogram analysis system is proposed to suggest transcatheter or surgical closure. Our DKS model provides interpretable and more accurate AI-assisted suggestions than the “black-box” binary classification. Though it can work fully automated, it also provides an intervention window for the experienced physicians. In the future, similar studies using deep learning algorithms shall be developed for size-sensitive treatments. To our knowledge, this is the first attempt to develop an AI-assisted strategy for CHD treatment planning (e.g., transcatheter or surgical closure suggestion) and occluder sizing. In addition, we collected and labeled a novel keypoint dataset in multiview echocardiograms for ASD, which enables the study of deep learning-based heart keypoint localization. By identifying the explicit clinical practice guidelines, our deep keypoint stadiometry algorithms were able to automatically propose the therapeutic plan, effectively reducing the workload of the clinicians.

## 4. Materials and Methods

### 4.1. Study Design and Datasets

The first stage of the retrospective study included 450 patients (184 males and 266 females, ages ranging from 5 months to 16 years, 4.19 ± 3.11 years) receiving ASD treatment from the Beijing Children's Hospital (BCH). To demonstrate the generality of our model, we evaluated it with another dataset with 129 patients from BCH with different sonographers (55 males and 74 females, ages ranging from 10 months to 16 years and 5 months, 5.29 ± 3.31 years). The detailed statistics of the collected patients are shown in Supplementary Table 1. According to the result, there are no significant differences in clinical features, which are estimated based on the average of clinicians' labeling between two datasets.

All of the collected patients have both the 2D and Doppler TTE [28] frames in three views, i.e., the view of parasternal short-axis (PSSAX) of the aorta, the view of subxiphoid long-axis (SXLAX) of two atria, and the view of apical four chamber (A4C). It is necessary and sufficient to derive a therapeutic plan from these views in clinical practice. The measuring scale is recorded along with each image as metadata.

The patient was placed in the supine position, and the chest was exposed for the echocardiogram. The imaging devices are PHILIPS iE33, IE ELITE, and EPIQ 7C, which have the frequency of the transducer between 3 and 8 MHz. The atrial septum defect was observed in the view of PSSAX of the aorta, SXLAX of two atria, and A4C. We measured the defect diameter, the distance of defect to the atrial wall in the view of PSSAX of the aorta; measured the defect diameter, the distance of defect to the superior and inferior vena cava in the view of SXLAX of two atria; and measured the defect diameter, the atrial septum length, the distance of defect to the atrial roof and the mitral annulus in the view of A4C. As preprocessing, we normalized all of the echocardiograms to the grayscale of 0-255, which further eliminated the interscanner and intersubject variations.

All patients' closure plans and the occluder size (i.e., waist diameter of the AMPLATZER™ Septal Occluder, from 8 mm to 32 mm) were confirmed by the final intraoperative diagnosis. All the keypoint labels were checked by two experienced clinicians. The study protocol was approved by the Ethics Committee of Beijing Children's Hospital.

### 4.2. “Black-box” Deep Learning Model as Baseline

**Figure 3:**
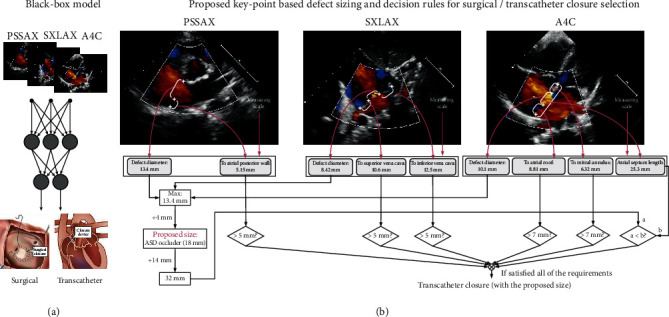
Illustration of our proposed frameworks for transcatheter or surgical closure suggestion of ASD with multiview Doppler TTE. (a) A “black-box” model and (b) a deep keypoint stadiometry (DKS) model. PSSAX: short for the aorta parasternal short-axis of the aorta, SXLAX is short for subxiphoid long-axis of two atria. The Boolean operations take the clinical guidance of AMPLATZER™ Septal Occluder as an example.

A possible solution to suggest the two closure methods is formulating the task as a binary classification task and applying the convolutional neural networks to map the three views to the class prediction (as shown in Figure 3 (a)). The measuring scale is also being concatenated to the fully connected layer to provide the scaling information. However, the network is nontransparent and hard to exploit the inherent causal relationship with a relatively weak class-level supervision. We refer to this solution as the “black-box” model, which utilizes a recent state-of-the-art network structure for multiview echocardiogram classification [20] (model is detailed in Supplementary Section 3.1). Notably, [20] focus on normal or patient diagnosis. The proposed baseline model is also novel to apply the DL classification model for treatment planning.

### 4.3. Deep Keypoint Stadiometry Model

Instead, we proposed to assist the intervention following the expert consensus for the interventional treatment of pediatric congenital heart disease with the AMPLATZER™ Septal Occluder, as shown in Figure 3 (b) (detailed in Supplementary Section 1.4) [29]. Our model has the potential to assist clinicians in formulating patient-specific closure plans and specifying the appropriate occluder size. We first automated the keypoint localization in each view to measure the distance between these points with the scale, which is a typical processing in clinical practice. Based on the stadiometry results, we explicitly followed the decision rules of the occluder with serial Boolean expressions to provide the interpretable therapeutic suggestion. We note that the Boolean expression consisted of the Boolean operators (e.g., OR, AND, and NOT) and conditional statements (e.g., >1), which produce the Boolean value of either true or false. Notably, the rules are varied for different occluders as detailed in their operation guidance. The size should match the length of the defect and surrounding rims, as detailed in Figure S.2. In Figure 3, we used AMPLATZER™ Septal Occluder as an example, which can be simply revised in the Boolean expressions of our DKS model without changing the keypoint localization parts.

The stacked hourglass network (SHN) has demonstrated effectiveness in human body keypoint detection [28]. However, SHN is sensitive to a specific scale in its pyramid-style networks and is not able to provide a coherent and robust response at all scales. Therefore, we have added layer-wise supervisions for all the deconvolutional layers. In addition, to efficiently integrate the anatomical structure of the ASD as prior to instructing our networks, we proposed to model the spatial connections of the keypoints in each view with an intermediate anatomical-aware loss. The priors of the anatomical structure are helpful for inferring the positions of the ambiguous points from clear and confidently localized points.

**Figure 4:**
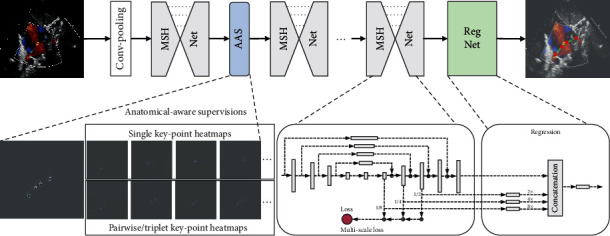
The detailed network structure for keypoint localization, which consist of MSHNet for rough localization, AAS for incorporating anatomical prior knowledge, and RegNet for final precise localization. MSHNet=multiscale hourglass network, RegNet=regression network.

Specifically, as shown in Figure 4, we constructed three novel submodules, i.e., multiscale hourglass network (MSHNet), anatomical-aware supervision (AAS) loss, and regression network (RegNet). The detailed network structure and loss are provided in Supplementary Section 3.2. In each cascaded MSHNet, we predict the rough position of the keypoint. Specifically, we extracted the feature maps in each deconvolutional layer with the spatial size of 1/8, 1/4, and 1/2 that of the input. Each feature map is transformed to a keypoint heat map with a 1 × 1 convolution, rendering the same number of channels as the number of keypoints. Therefore, each channel corresponds to a keypoint. We downsampled the ground truth heat map to fit the extracted heat maps at different scales to make the comparisons. The mean square error (MSE) for each point at all layers was calculated and added up as the multiscale loss. In AAS, we propose to incorporate the relationship between these keypoints into consideration to help the localization. Other than the single pointwise loss, we also calculated the MSE loss w.r.t. pairwise and triplet keypoint heat maps to explore the high-order associations of these points. The cooccurrence of the keypoint pair or triplet can better capture the adjacency and the association among the points in each view. Lastly, we used a fully convolutional RegNet to make the final prediction of keypoint locations. The heat maps in multiple scales are taken as inputs and matched to the ground truth keypoint at the corresponding scales. Therefore, our RegNet can fuse the heat maps at different scales for localization refinement.

With the predicted keypoints and the scale of the echocardiogram imaging, we were able to measure the length of the defect area and the surrounding rims, as shown in Figure 3. We compared these lengths with their corresponding thresholds, which are defined by the typical size of the ASD occluder. If all the conditions are satisfied, the transcatheter closure shall be recommended. Otherwise, a surgical closure is necessary. For the case of transcatheter closure, the size of the occluder can be the maximum defect diameter of three views plus 4 mm. Hyperparameter optimization was set up to maximize peak training PCK. Testing performance was not computed until reporting the final results. Best performing architectures were then tested on the testing set. We note that both the DKS and “black-box” models are tuned using the same protocol. The training protocol is detailed in Supplementary Section 4.

### 4.4. Evaluations

For within-center evaluation, we used 330 randomly selected patients (180 transcatheter closures and 150 surgical closures) for training, 30 patients (20 transcatheter closures and 10 surgical closures) were used for hyperparameter validations, and the remaining 90 patients (50 transcatheter closures and 40 surgical closures) for testing following a subject-independent manner. The second evaluation dataset included 129 patients (110 transcatheter closures and 19 surgical closures). All the methods are trained and tested five times to report the standard deviation. Since not all the 2D and Doppler images are pixel-wise aligned, we split them for independent processing and compared their performance. The evaluation metrics are detailed in Supplementary Section 5.

With the scale, it is possible to measure the absolute distance between these points. The first objective of our study was the percentage of correct keypoint (PCK) metric of keypoint localization in three views [30]. Notably, PCK is a regression metric for evaluation point localization performance, which is only applicable to the DKS model. Specifically, our PCK was calculated as the percentage of disparities between the detected keypoints w.r.t. the ground truth after normalization against a fraction of the measuring scale length.

Then, we predicted the therapeutic plan following the Boolean expressions. We evaluated the accuracy, F1 score, sensitivity, and specificity for the classification of transcatheter or surgical closure suggestions. F1 score is the harmonic mean of the precision and recall, i.e., 2 × precision × recall/(precision + recall). We noted that the DKS model had no threshold for binary classification in contrast to the “black-box” model. Therefore, the receiver operating characteristic is not applicable here.

The suggested ASD occluder size was also predicted for the transcatheter closure cases. We adopted the metrics of mean absolute error (MAE) and the quadratic weighted kappa (QWK) commonly used in the ordinal classification task [31] (detailed in Supplementary Section 5). MAE and QWK can punish the misclassification proportional to the distance between the predicted label of the network and the ground truth label. Therefore, predicting the case of 16 mm as 14 mm can be more acceptable than predicting it as 10 mm. The detailed metric formulations are provided in Supplementary Section 5. The metrics of MAE and QWK can reflect the discrepancy between the predicted size and size label. We note that the MAE measures the difference between the length predicted by the model and the ground truth. Therefore, the smaller MAE indicates the more accurate predictions. In contrast, the QWK measures the consistency of the model results and labels, with a value of 1 for a perfect match and 0 for a no match at all.

## Figures and Tables

**Table 1a:** (a) Within-center evaluation set

Methods	Modality	PSSAX of the aorta (three points)	SXLAX of two atria (four points)	A4C (four points)	Average
DKS	Doppler	0.9858 ± 0.0034	0.9674 ± 0.0030	0.9735 ± 0.0035	0.9746 ± 0.0033
DKS w/o MSH	Doppler	0.9783 ± 0.0030	0.9511 ± 0.0035	0.9635 ± 0.0037	0.9630 ± 0.0036
DKS w/o AAS	Doppler	0.9762 ± 0.0034	0.9534 ± 0.0032	0.9621 ± 0.0038	0.9628 ± 0.0035
DKS w/o MSH&AAS	Doppler	0.9691 ± 0.0035	0.9472 ± 0.0036	0.9565 ± 0.0037	0.9566 ± 0.0036
DKS using 2D	2D	0.8938 ± 0.0041	0.8567 ± 0.0037	0.8846 ± 0.0040	0.8769 ± 0.0039

**Table 1b:** (b) Cross-center evaluation set

Methods	Modality	PSSAX of the aorta (three points)	SXLAX of two atria (four points)	A4C (four points)	Average
DKS	Doppler	0.9754 ± 0.0037	0.9633 ± 0.0030	0.9694 ± 0.0035	0.9688 ± 0.0034
DKS w/o MSH	Doppler	0.9705 ± 0.0045	0.9525 ± 0.0035	0.9601 ± 0.0037	0.9602 ± 0.0038
DKS w/o AAS	Doppler	0.9682 ± 0.0040	0.9498 ± 0.0032	0.9579 ± 0.0038	0.9578 ± 0.0036
DKS w/o MSH&AAS	Doppler	0.9617 ± 0.0042	0.9462 ± 0.0036	0.9513 ± 0.0037	0.9523 ± 0.0038

**Table 2a:** (a) Within-center evaluation set

Methods	Modality	ACC	F1	Sensitivity	Specificity	*p* value^∗^
Black-box	Doppler	0.7646 ± 0.0068	0.7718 ± 0.0055	0.7651 ± 0.0063	0.7432 ± 0.0059	<0.0001
DKS	Doppler	0.9425 ± 0.0052	0.9496 ± 0.0047	0.9404 ± 0.0056	0.9528 ± 0.0045	Ref
DKS w/o MS	Doppler	0.9312 ± 0.0057	0.9318 ± 0.0050	0.9254 ± 0.0058	0.9456 ± 0.0047	0.042
DKS w/o SL	Doppler	0.9344 ± 0.0051	0.9345 ± 0.0049	0.9261 ± 0.0052	0.9417 ± 0.0042	0.037
DKS w/o MSH&AAS	Doppler	0.9293 ± 0.0047	0.9249 ± 0.0055	0.9204 ± 0.0047	0.9375 ± 0.0051	0.018
DKS using 2D	2D	0.8781 ± 0.0053	0.8894 ± 0.0058	0.8823 ± 0.0054	0.8758 ± 0.0048	<0.0001

**Table 2b:** (b) Cross-center evaluation set

Methods	Modality	ACC	F1	Sensitivity	Specificity	*p* value
Black-box	Doppler	0.7637 ± 0.0054	0.7658 ± 0.0052	0.7626 ± 0.0058	0.7432 ± 0.0060	<0.0001
DKS	Doppler	0.9385 ± 0.0050	0.9416 ± 0.0047	0.9373 ± 0.0053	0.9507 ± 0.0048	Ref
DKS w/o MS	Doppler	0.9265 ± 0.0052	0.9274 ± 0.005	0.9205 ± 0.0055	0.9461 ± 0.0050	0.046
DKS w/o SL	Doppler	0.9213 ± 0.0048	0.9229 ± 0.0050	0.9241 ± 0.0052	0.9398 ± 0.0045	0.035
DKS w/o MSH&AAS	Doppler	0.9192 ± 0.0053	0.9205 ± 0.0051	0.9175 ± 0.0053	0.9352 ± 0.0049	0.021

## Data Availability

All data collected for the study are not publicly available for download regarding patient confidentiality and consent. However, the corresponding authors can be contacted for academic inquiry.

## Abbreviations

ASD: Atrial septal defect
CHD: Congenital heart disease
AI: Artificial intelligence
TTE: Transthoracic echocardiogram
TEE: Transesophageal echocardiogram
PSSAX: Parasternal short-axis
SXLAX: Subxiphoid long-axis
A4C: Apical four chamber
DSK: Deep keypoint stadiometry
SHN: Stacked hourglass network
MSHNet: Multiscale hourglass network
AAS: Anatomical-aware supervision
RegNet: Regression network
MSE: Mean square error
QWK: Quadratic weighted kappa
PCK: Percentage of correct keypoint.

## Supplementary Materials

Supplementary Materials The supplementary incorporates the task background, data collection, model details, evaluation metrics, and failure case analysis. Figure S1: the accuracy concerning the number of training epochs for the “black-box” model and deep keypoint stadiometry model. Figure S2: comparison of the occluder size prediction with MAE (the smaller, the better) and QWK (the larger, the better) metrics. Supplementary Table 1: the statistics of the clinical characteristics of collected ASD patients.
