# Supplementary material for: Assessment of Transcatheter or Surgical Closure of Atrial Septal Defect using Interpretable Deep Keypoint Stadiometry
Source: Research (Wash D C). 2022 Oct 21;2022:9790653. doi: 10.34133/2022/9790653 (PMC9620637; doi:10.34133/2022/9790653)
Supplement: Supplementary Materials — The supplementary incorporates the task background, data collection, model details, evaluation metrics, and failure case analysis. Figure S1: the accuracy concerning the number of training epochs for the “black-box” model and deep keypoint stadiometry model. Figure S2: comparison of the occluder size prediction with MAE (the smaller, the better) and QWK (the larger, the better) metrics. Supplementary Table 1: the statistics of the clinical characteristics of collected ASD patients. [file 9790653.f1.zip › Research(supplymentary)/images/picture5.pdf]

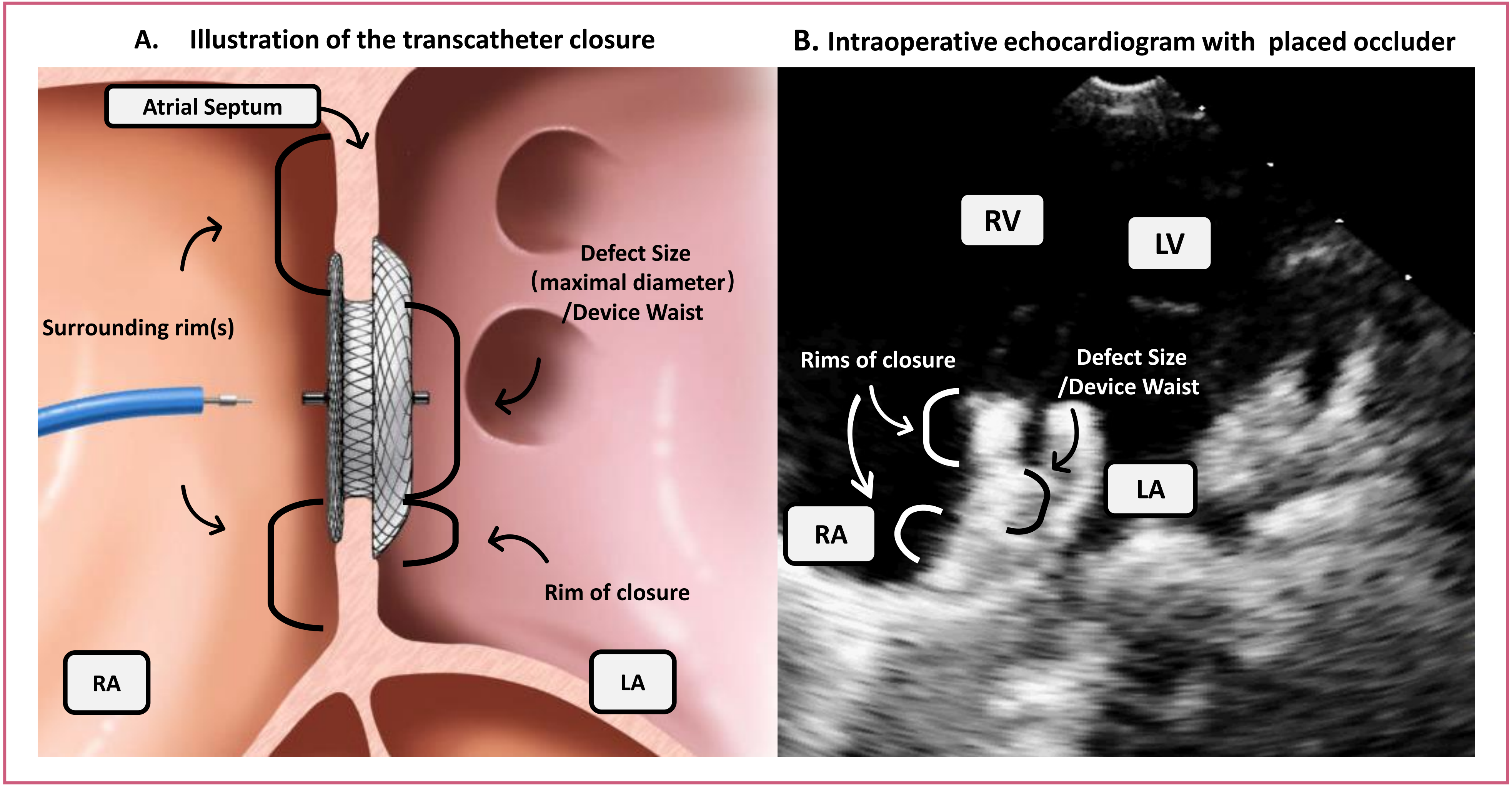
A. Illustration of the transcatheter closure
B. Intraoperative echocardiogram with placed occluder
Atrial Septum
Defect Size
(maximal diameter)
/Device Waist
Surrounding rim(s)
Rim of closure
RA
LA
RV
LV
Rims of closure
Defect Size
/Device Waist
LA
RA
