# Supplementary material for: Assessment of Transcatheter or Surgical Closure of Atrial Septal Defect using Interpretable Deep Keypoint Stadiometry
Source: Research (Wash D C). 2022 Oct 21;2022:9790653. doi: 10.34133/2022/9790653 (PMC9620637; doi:10.34133/2022/9790653)
Supplement: Supplementary Materials — The supplementary incorporates the task background, data collection, model details, evaluation metrics, and failure case analysis. Figure S1: the accuracy concerning the number of training epochs for the “black-box” model and deep keypoint stadiometry model. Figure S2: comparison of the occluder size prediction with MAE (the smaller, the better) and QWK (the larger, the better) metrics. Supplementary Table 1: the statistics of the clinical characteristics of collected ASD patients. [file 9790653.f1.zip › Research__supplymentary.pdf]

Assessment of Transcatheter or Surgical Closure of Atrial Septal Defect using Interpretable Deep Keypoint Stadiometry

Supplementary Material

Jing Wang, Ph.D[a,1], Wanqing Xie, Ph.D[d,e,1], Qun Wu, MA.Sc[b], Fangyun Wang, M.D.[b], Pei Li, MA.Sc[b], Bo Fan, B.S.[a], Xin Zhang, M.D.[b,*], Binbin Wang, Ph.D[f,g,*], Xiaofeng Liu, Ph.D[c,*]

[a]*School of Basic Medical Sciences, Capital Medical University, Beijing, 10069, China*

[b]*Heart Center, Beijing Children's Hospital, Capital Medical University, National Center for Children's Health, Beijing, 10045, China*

[c]*Gordon Center for Medical Imaging, Harvard Medical School and Massachusetts General Hospital, Boston, MA, 02114, United States*

[d]*Department of Intelligent Medical Engineering, School of Biomedical Engineering, Anhui Medical University, Hefei, 230032, PR China*

[e]*Beth Israel Deaconess Medical Center, Harvard Medical School, Harvard University, Boston, MA, 02215, United States*

[f]*Center for Genetics, National Research Institute for Family Planning, Beijing, 100730, China*

[g]*Graduated school, Peking Union Medical College, Beijing, 100730, China*

---

*Corresponding authors: Xin Zhang (Tel:+86 10 59616161), Binbin Wang (Tel: +86 10 62173443; Fax: +86 10 62179086) and Xiaofeng Liu (Tel: +1 4123307754)

Email addresses: zhangxin1651@163.com (Xin Zhang, M.D.), wbbahu@163.com (Binbin Wang, Ph.D), xliu61@mgh.harvard.edu (Xiaofeng Liu, Ph.D)

[1]These authors contributed equally to this work.

Contents

1 Background of the task **3**

1.1 What is an atrial septal defect? . . . 3

1.2 How to repair atrial septal defect? . . . 3

1.3 Occluder for transcatheter closure . . . 4

1.4 Which condition is suitable for transcatheter ASD closure? . . . 4

2 Data Collection **6**

2.1 Study Population . . . 6

2.2 Collection Protocol of Cardiac Ultrasound Image Data . . . 7

2.3 Measuring Scale . . . 8

2.4 Example of a Subject . . . 9

2.5 Pre-processing . . . 12

3 Details of Model and Loss Function **13**

3.1 Black-box Model . . . 13

3.2 Deep Keypoint Stadiometry . . . 14

3.2.1 Multi-scale Hourglass Networks . . . 15

3.2.2 Multi-Scale Regression Network . . . 16

3.2.3 Anatomical-aware Loss . . . 16

4 Training Details **18**

4.1 Platform . . . 18

4.2 Hyperparameter Search . . . 18

4.3 Training Setup . . . 18

4.4 Class Balancing in “Black-box" Model . . . 18

5 Evaluation Metrics **19**

6 Failure Analysis **20**

1 Background of the task

1.1 What is an atrial septal defect?

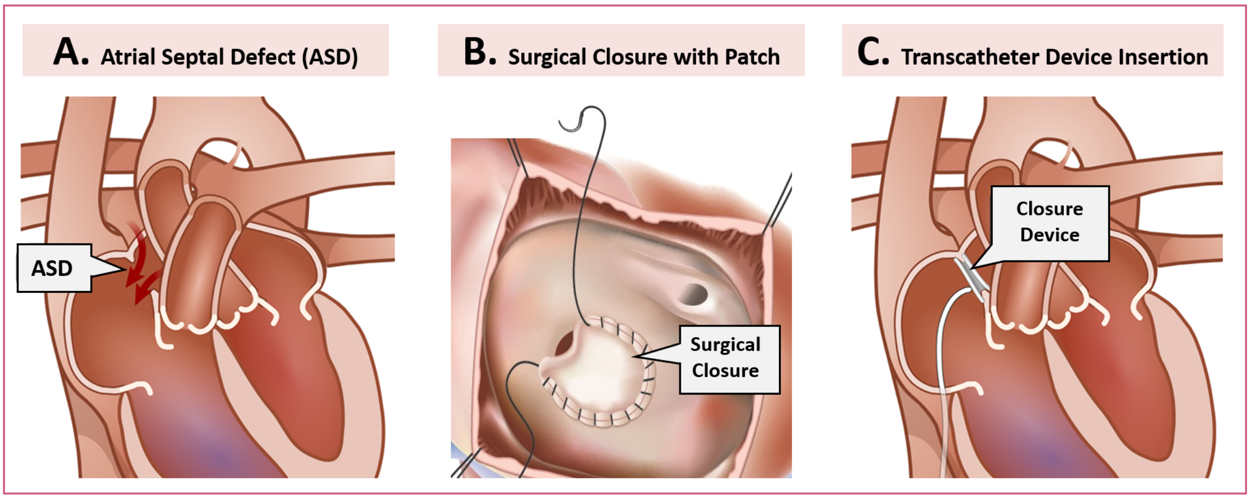

Figure 1. Illustration of the ASD (A), and its repair plan: transcatheter closure (B) and surgical closure (C).

An atrial septal defect (ASD) is a hole in the wall that connects the heart's two upper chambers. (as shown in Supplementary Figure 1 A).[2] This wall is called the atrial septum.

The left side of the heart normally only pumps blood to the body, while the right side only pumps blood to the lungs. However, blood will flow from the left atrium to the right atrium and out through the lung arteries in a child with ASD. Since more blood than expected is going from the right ventricle to the lungs, the right side of the heart has to function harder.[1]

1.2 How to repair atrial septal defect?

Several decades ago, surgery was the only treatment option for heart abnormalities. However, thanks to surgical advancements and impressive advances in medical science, many systemic heart diseases have been successfully repaired with catheterization in recent years, e.g., ostium secundum ASD, ventricular septal defect, patent foramen ovale, patent ductus arteriosus, mitral stenosis, and aortic stenosis. One of the first CHD to be treated using a transcatheter approach was ASD. Recently, it has been considered the standard solution in many cases.[2,3]

For some heart defects such as ASD, PDA, and VSD, to close a hole in the heart, a small closure mechanism may be implanted (as shown in Supplementary Figure 1 B). In certain circumstances, this is sufficient to resolve the problem.

Transcatheter closure of ASD has been used successfully in both children and adults for many years. Accurate measuring the rims and the defect diameter of ASD is important for the success of transcatheter ASD closure.

The balloon sizing technique has been used in the past, using fluoroscopy as a guide.[4,5] However, over the last several years, transthoracic echocardiogram (TTE) guidance alone has been increasingly used in place of fluoroscopy.[6,7] If the ASD is too large or in the improper place for the occluder to seal, surgical closure will be suggested (as shown in Supplementary Figure 1 C).

[2]https://www.aboutkidshealth.ca/Article?contentid=1607&language=English

1.3 Occluder for transcatheter closure

Closure products come in a variety of shapes and sizes. When in place, an occluder, for example, resembles two opened umbrellas.[3] The occluder is held in place by spring arms that keep blood from passing into the cavity (as shown in Supplementary Figure 2). The arms remain closed until the device is in the correct location. The umbrella-like structure is then opened.

It is important to match the diameter and the dimensions of various rims of ASD and the size of the occluder. If the size is not well matched, some adverse events such as device embolization/migration, cardiac erosion, or cardiac arrhythmia will accidentally happen.

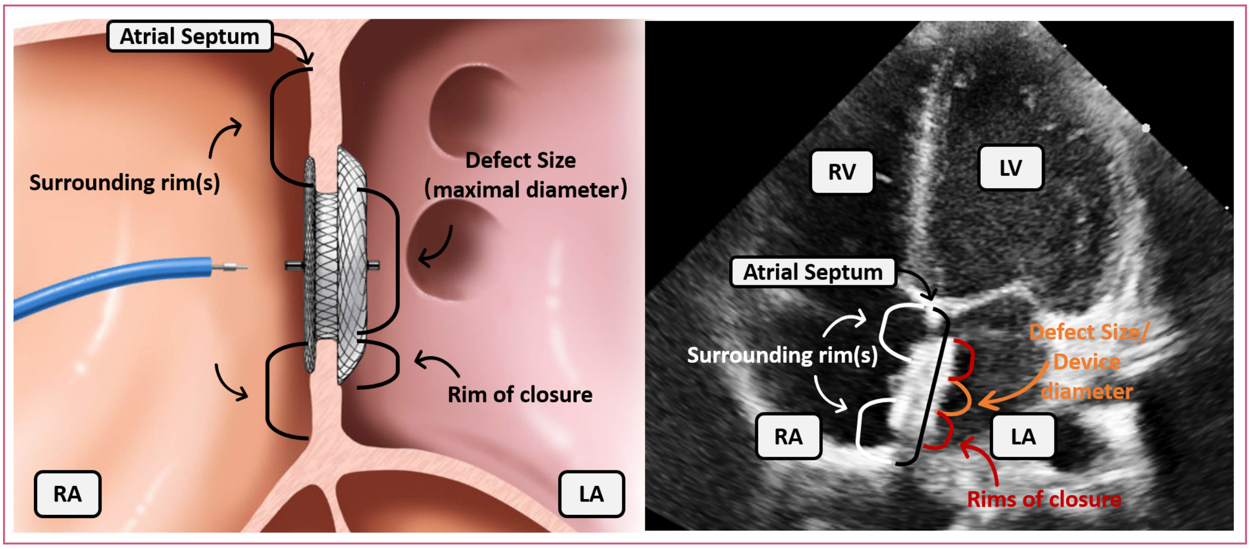

Figure 2. Illustration of the ASD occulder in transcatheter closure. (take the Amplatzer™ Septal Occluder as an example)

1.4 Which condition is suitable for transcatheter ASD closure?

The expert consensus for the interventional treatment of pediatric congenital heart disease in China defined four classifications of recommendations for transcatheter device closure of secundum ASD.[8] Among them, class I and class IIa are recommended for transcatheter closure in the clinic, as follows:

Class I: Conditions for evidence and/or general agreements that the given procedure is beneficial and effective.

1. Age$\geq$2 years;
2. Secundum ASD with a hemodynamically significant shunt (ASD minimum diameter$\geq$5 mm);
3. The distance between ASD and coronary sinus (CS rim)$\geq$5 mm; the distance between ASD and the superior vena cava (SVC rim)$\geq$5 mm; the distance between ASD and the inferior vena cava (IVC rim)$\geq$5 mm; the distance between ASD and the pulmonary vein (PV rim)$\geq$5 mm;
4. The distance between ASD and the atrioventricular valves$\geq$7 mm;
5. Atrial septum length > Left atrial disc diameter of the device;
6. Without other cardiac malformations.

[3] https://www.aboutkidshealth.ca/Article?contentid=1670&language=English

Class IIa: Conditions for evidence and/or general agreements that the given procedure is useful or effective.

1. Age<2 years, secundum ASD with hemodynamically significant shunt and suitable anatomic structures;

2. Adequate rims of surrounding tissues except for anterior rim (the distance between ASD and the anterior atrial walls), secundum atrial septal defect with a hemodynamically significant shunt;

3. Multi-hole secundum atrial septal defect with a hemodynamically significant shunt.

2 Data Collection

2.1 Study Population

In this retrospective study, we collected 450 patients (184 males and 266 females, ages ranging from 5 months to 16 years, 4·19±3·11 years) who underwent the operation on ASD from the Beijing Children's Hospital.

We used 360 randomly selected patients (200 transcatheter closure and 160 surgical closure) for training and the remaining 90 patients (50 transcatheter closure and 40 surgical closure) for testing. The number of each occluder size in the transcatheter subset is shown in Supplementary Table 1. All patients' closure plans and the occluder size (i.e., occluder waist diameter, from 8 mm to 32 mm) were confirmed by the final intraoperative diagnosis.

To validate our model, we built another dataset, including 129 patients, 110 of them were treated with transcatheter closure (48 males and 62 females, ages ranging from 1 year 1 month to 16 years 5 months, 6·36±3·38 years), and 19 of them were treated with surgical closure (7 males and 12 females, ages ranged from 10 months to 8 years 11 months, 3·52±2·45 years).

Table 1. The common information of occluder and the distribution of occluder size in transcatheter subset. (take the Amplatzer™ Septal Occluder as an example)

Occluder Waist Diameter (mm)	8	10	11	12	13	14	15	16	17	18	20	22	24	25	26	28	30	32
Right Atrial Disc Diameter (mm)	16	18	21	22	23	24	25	26	27	28	30	32	34	35	36	38	40	42
Left Atrial Disc Diameter (mm)	20	22	25	26	27	28	29	30	31	32	34	36	38	39	40	42	44	46
Number of participants	13	28	4	63	10	51	4	22	2	11	15	7	7	1	3	2	4	3

2.2 Collection Protocol of Cardiac Ultrasound Image Data

For the echocardiogram, the patient was seated in a supine posture with his chest exposed. The imaging devices are PHILIPS iE 33, iE Elite, and EPIQ 7C, which have the frequency of the transducer between 3-8 MHz. The defect of the atrial septum was observed in the view of the parasternal short-axis (PSSAX) of the aorta, the view of the subxiphoid long-axis (SXLAX) of two atria, and the view of the apical four chambers (A4C).

All of the collected patients have both the 2D and Doppler echocardiogram videos in three views, i.e., the view of PSSAX of the aorta, the view of SXLAX of two atria, and the view of A4C. The clinicians selected a key frame in each video, which forms the image-based sub-datasets of 2D or Doppler TTE.

The Doppler echocardiogram has been developed for measuring and accessing the blood flow within the chambers and valves of the heart. The amount of blood pumping out of each heartbeat is a measure of how well the heart is working. Doppler can also sense decreased blood pressure inside the heart, which may mean a fault with one or more of the four heart valves or the walls of the heart.[9]

All of the key-point labels are checked by two experienced doctors. We measured the defect diameter, the distance of defect to the atrial wall in the view of PSSAX of the aorta; measured the defect diameter, the distance of defect to the superior and inferior vena cava in the view of SXLAX of two atria; measured the defect diameter, the atrial septum length, the distance of defect to the atrial roof and the mitral annulus in the view of A4C.

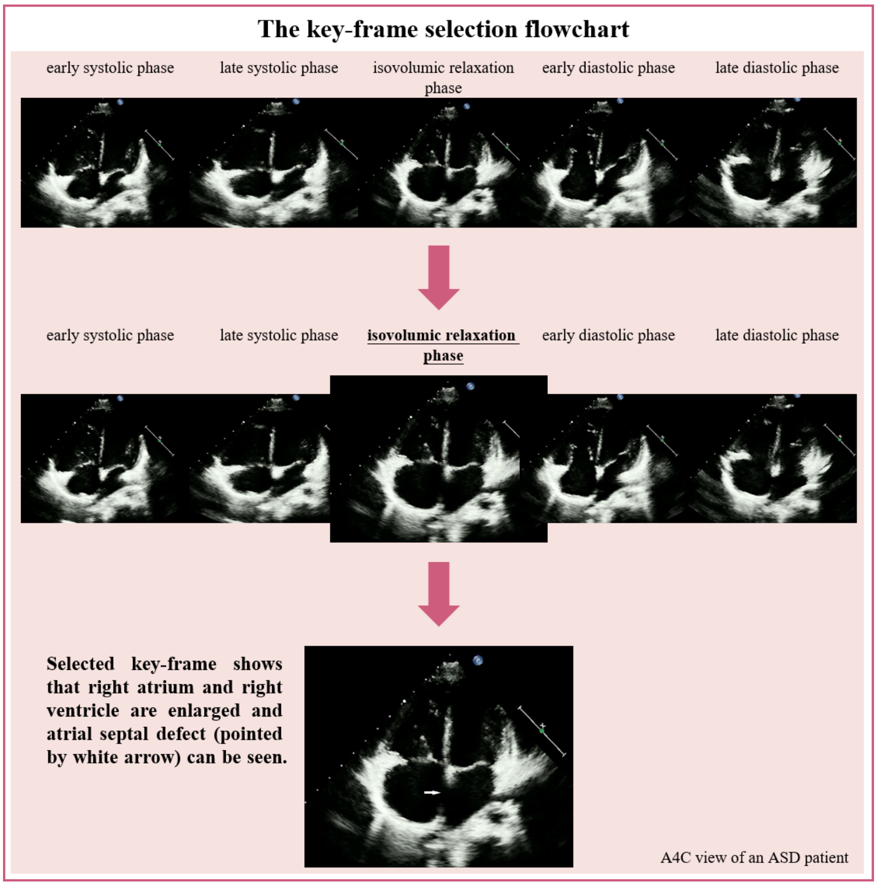

Figure 3. Illustration of the key-frame selection. We use A4C view as an example.

2.3 Measuring Scale

The measuring scale is recorded along with each echocardiogram frame as metadata. In addition, in the system, the measuring scale is shown along with the echocardiogram. As shown in Supplementary Figure 4, the measuring scale between two consecutive dots indicates the length of 1 cm.

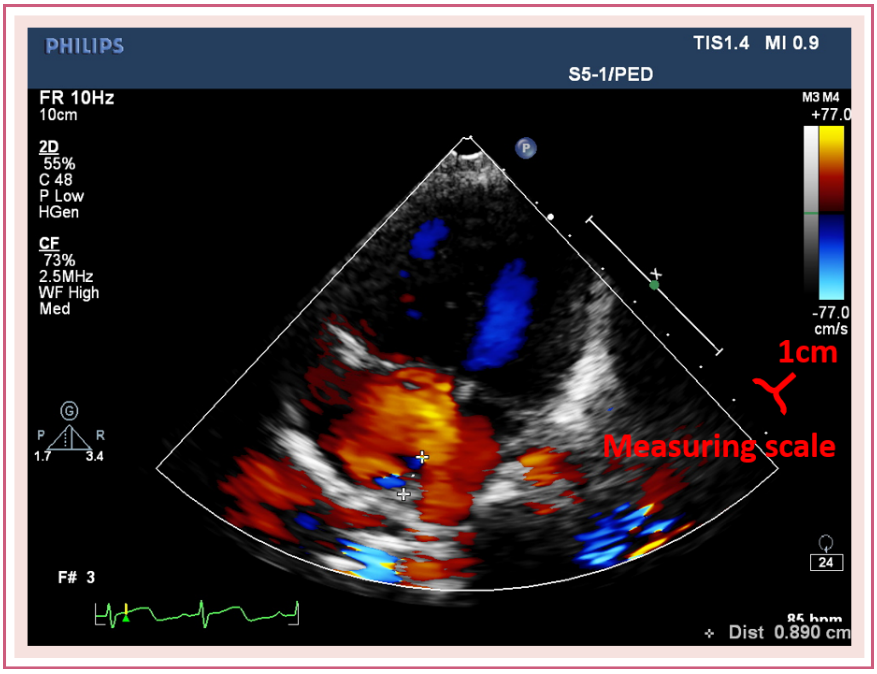

Figure 4. Illustration of the measuring scale.

2.4 Example of a Subject

In Supplementary Figure 5, we show a subject with both 2D and Doppler echocardiograms. We note that not all of the 2D and Doppler images are pixel-wise aligned. Therefore, we split them for independent processing and compared their performance. In Supplementary Figure 6 and Figure 7, we show the key-point labeling of 2D and Doppler echocardiograms in the view of PSSAX respectively.

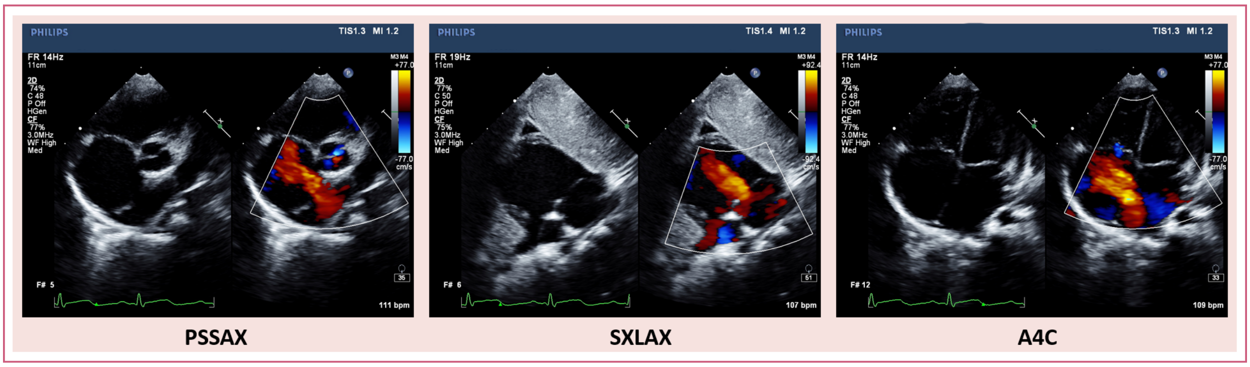

Figure 5. Illustration of a subject with 2D and Doppler echocardiograms in three views. PSSAX: short for PSSAX of the aorta; SXLAX: short for SXLAX of two atria

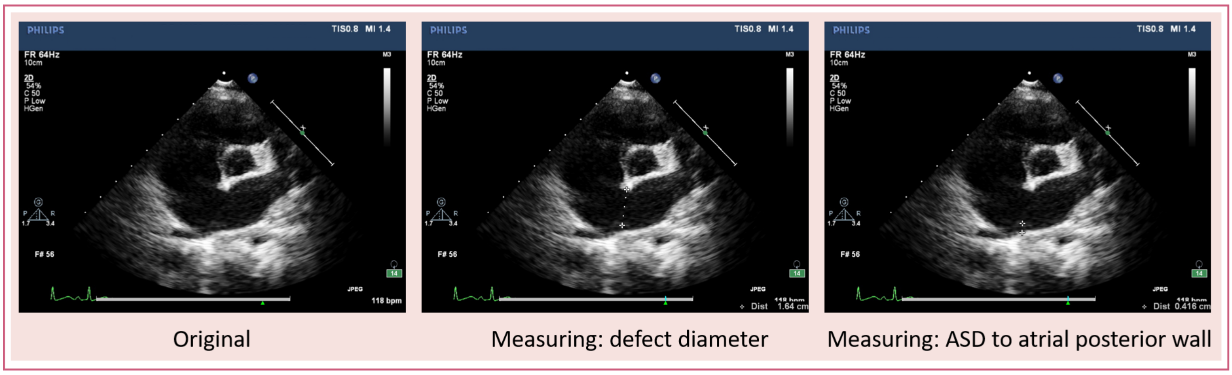

Figure 6. The labeling of 2D echocardiogram in the view of the view of PSSAX of the aorta

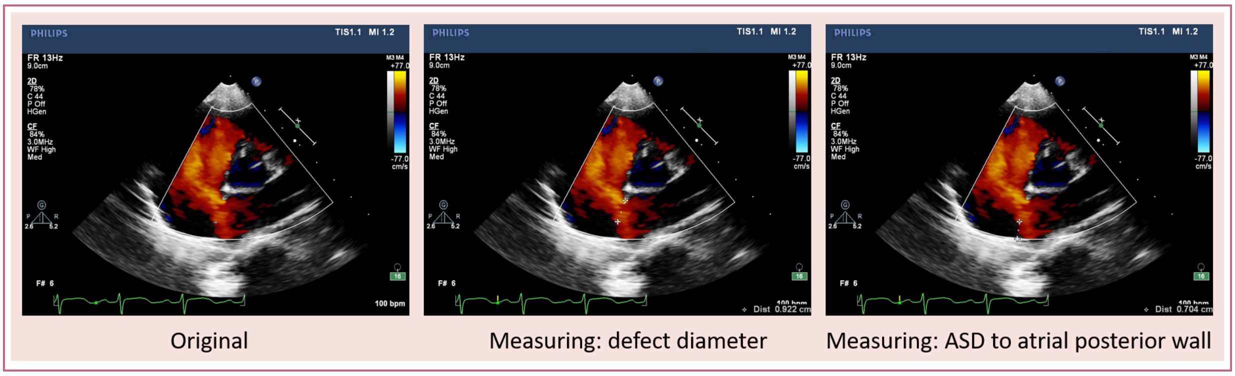

Figure 7. The labeling of Doppler echocardiogram in the view of PSSAX of the aorta

In Supplementary Figure 8 and 9, we show the key-point labeling of 2D and Doppler echocardiograms in the view of SXLAX of two atria.

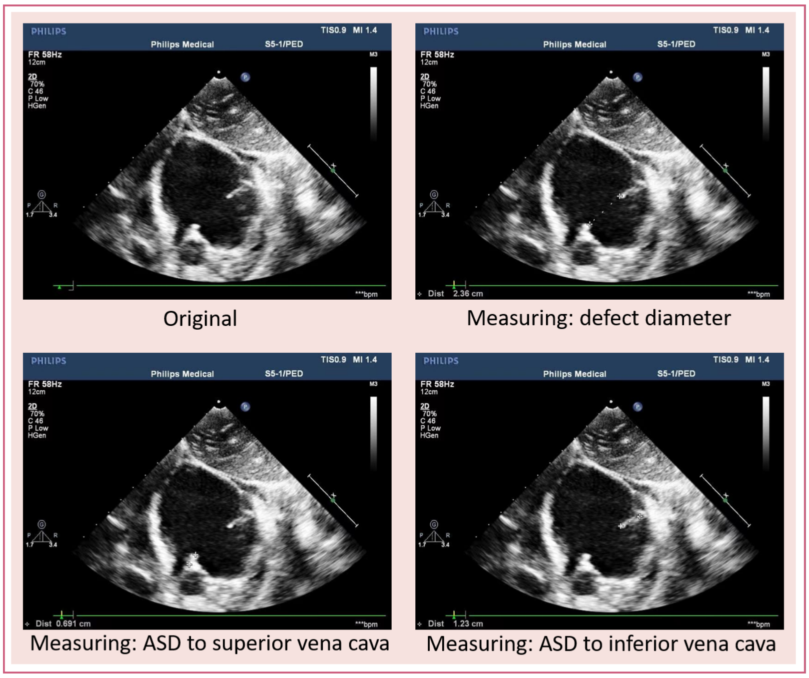

Figure 8. The labeling of 2D echocardiogram in the view of SXLAX of two atria.

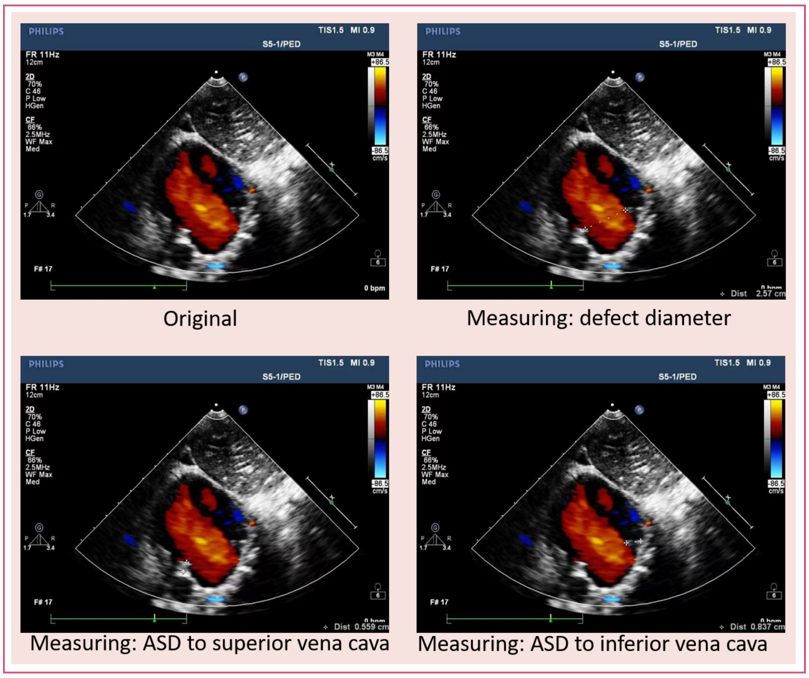

Figure 9. The labeling of Doppler echocardiogram in the view of SXLAX of two atria.

In Supplementary Figure 10 and Figure 11, we show the key-point labeling of 2D and Doppler echocardiograms in the view of A4C respectively.

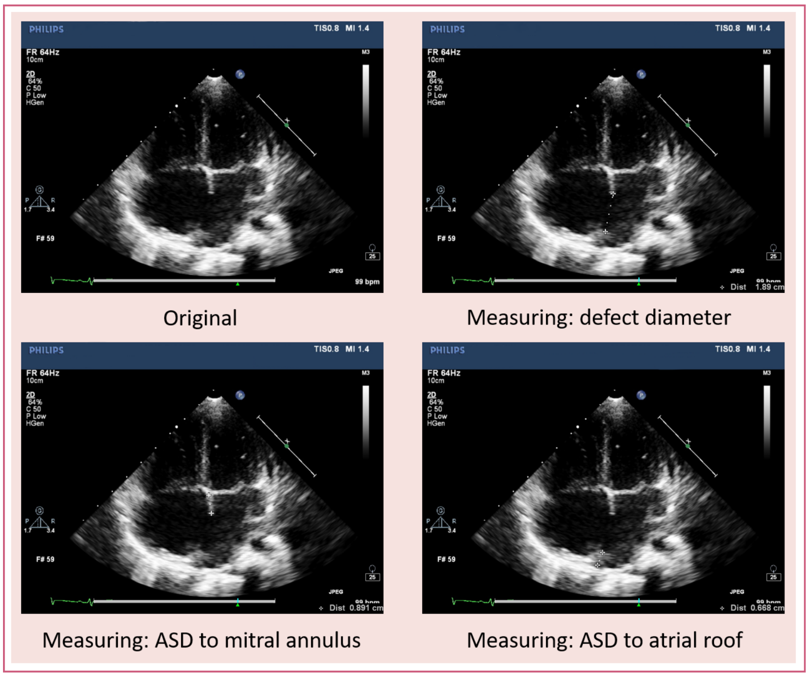

Figure 10. The labeling of 2D echocardiogram in the view of A4C.

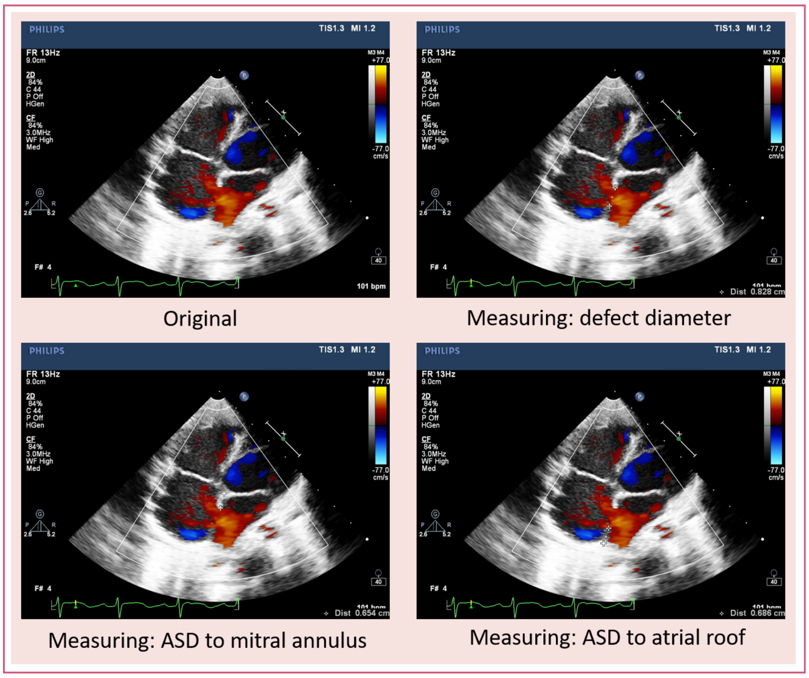

Figure 11. The labeling of Doppler echocardiogram in the view of A4C.

Each labeled keypoint is encoded as a two-dimensional vector (x, y) as shown in Supplementary Figure 12.

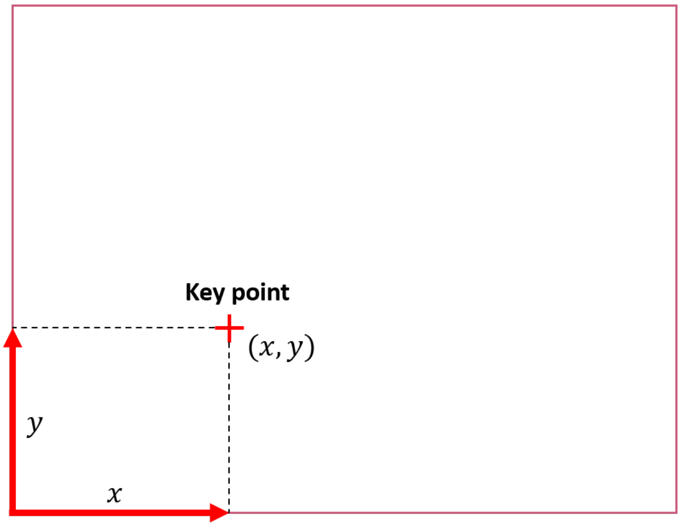

Figure 12. Illustration of the two-dimensional label of the keypoint.

2.5 Pre-processing

A mask is intended to cover these aspects since the captured echocardiogram images are circular sectors, and some of the surrounding labels cannot be removed by rectangular cropping.

To match the input size of the conventional convolutional neural networks, the masked ROI is rescaled to 128×128. In addition, we also adjust the measuring scale accordingly. The flow chart of pre-processing is shown in Supplementary Figure 13. We use the same pre-processing for the image of each view and concatenate these three images following fixed order: PSSAX of the aorta, SXLAX of two atria, and A4C. We note that each view only has one image in our 2D/doppler echocardiogram dataset.

We note that the original file of both 2D and Doppler echocardiograms has three channels, i.e., the pre-processed size is $128 \times 128 \times 3$. To keep the network structure consistent for these two modalities, we do not convert the 2D echocardiograms to a single channel.

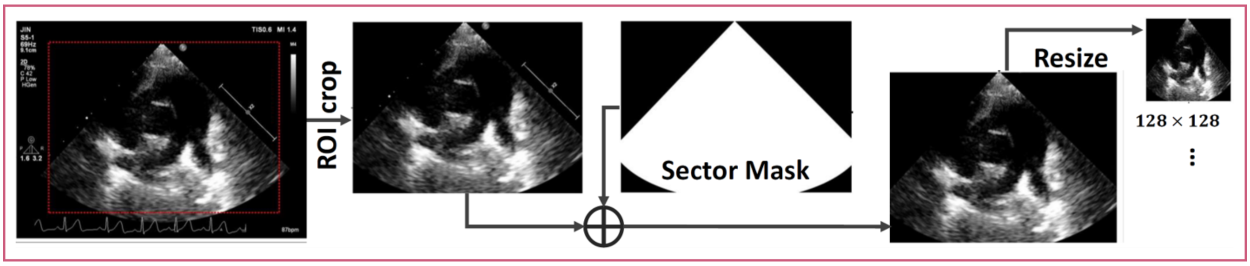

Figure 13. Prepossessing of the echocardiograms.

3 Details of Model and Loss Function

3.1 Black-box Model

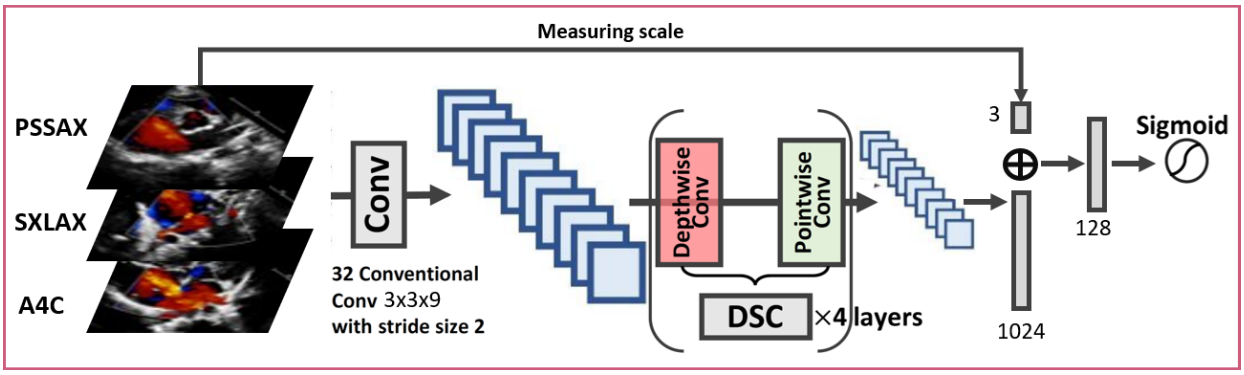

Figure 14. The network structure of the "black-box" classification model. PSSAX: short for PSSAX of the aorta; SXLAX: short for SXLAX of two atria

Table 2. The detailed structure of our "black-box" classification model.

Input Size	Type / Stride	Filter Shape
$128 \times 128 \times 9$	Conv / s2	32 kernels of $3 \times 3 \times 9$
$64 \times 64 \times 32$	Conv dw / s1	32 kernels of 3×3 dw
$64 \times 64 \times 32$	Conv pw / s1	64 kernels of$1 \times 1 \times 32$ pw
$64 \times 64 \times 64$	Conv dw / s2	64 kernels of3×3 dw
$32 \times 32 \times 64$	Conv pw / s1	128 kernels of$1 \times 1 \times 64$ pw
$32 \times 32 \times 128$	Conv dw / s2	128 kernels of 3×3 dw
$16 \times 16 \times 128$	Conv pw / s1	128 kernels of $1 \times 1 \times 128$ pw
$16 \times 16 \times 128$	Conv dw / s2	128 kernels of 3×3 dw
$8 \times 8 \times 128$	Conv pw / s1	128 kernels of $1 \times 1 \times 128$ pw
$8 \times 8 \times 128$	Flatten	N/A
8192	FC1	1024
1024 + 3	FC2	128
128	Classifier	Sigmoid

The "black-box" classification model is adapted from our multi-view Echocardiograms CHD diagnosis model.[10]

The input of our system is three-view echocardiograms and their corresponding measuring scale, which is sufficient for the classification of closure plans. The multi-view inputs provide complementary information for the decision-making, while also introducing the challenge of information fusion.

Following our previous multi-view echocardiograms analyzing framework,[10] the three echocardiograms are concatenated following a fixed sequence and form a cubic with the size of $128 \times 128 \times 9$. We note that each echocardiogram has 3 channels. Then, the cubic is processed by the network as shown in Supplementary Figure 14.

Following our previous work,[10] the Depthwise Separable Convolution (DSC)[11] is used as our backbone. Each DSC block consists of a depthwise convolution and a pointwise convolution. After a few DSC layers, the feature maps will be flattened as the feature vector that will be processed by two fully connected layers with sizes 1024 and 128, respectively. Moreover, the measuring scale is concatenated to the 1024-dim feature. The sigmoid unit outputs a scalar between 0 to 1, which indicates the probability. The detailed network structure is given in Supplementary Table 2, where dw denotes the depth-wise convolution with size $H \times W$, and keep the same for N channels. pw denotes the

pointwise convolution with size $1 \times 1 \times N$. We note that the first layer uses the conventional convolution with stride size two as in MobileNets.[11]

The measuring scale of each view is encoded as a scalar which is set to the absolute length of the width of the input image. The 3-dim measuring scale vector is concatenated to the 1024-dim feature for the second fully connected layer to provide the necessary scaling information.

We set the threshold of sigmoid output as 0·5. For the ground truth label, we encode transcatheter closure as one and surgical closure as zero. With the “black-box", it is not able to utilize the fine-grained supervision with keypoints.

3.2 Deep Keypoint Stadiometry

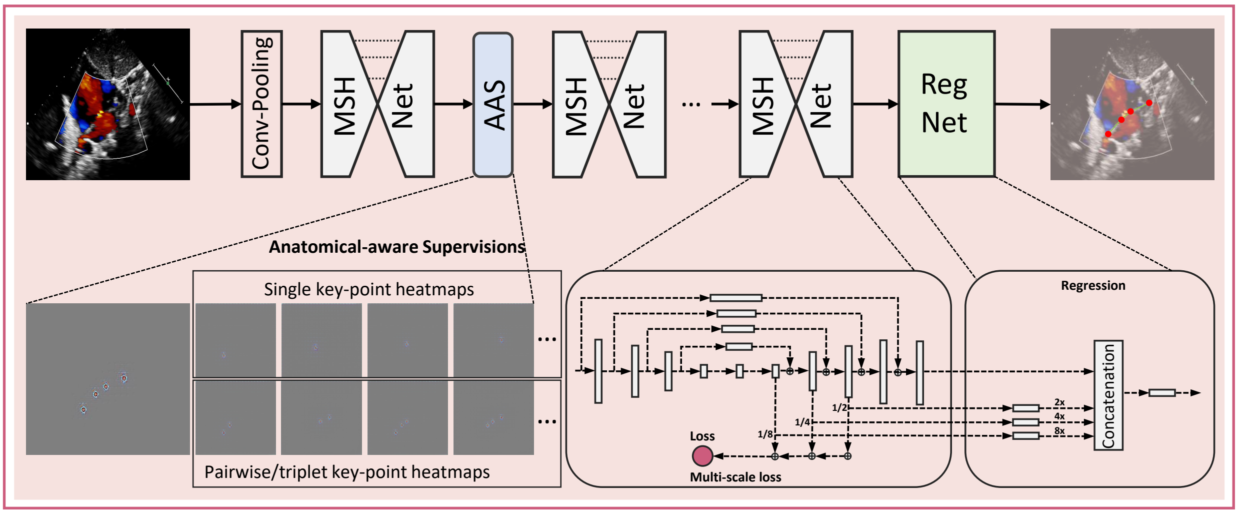

Figure 15. The detailed network structure for key-point localization. MSHNet: multi-scale hourglass networks, RegNet: regression network.

As shown in Supplementary Figure 15, our deep keypoint stadiometry network consists of two types of subnetworks, i.e., multi-scale hourglass networks (MSHNet) and regression networks (RegNet)

To extract the feature, we stacked several MSHNet as the stacked hourglass networks (SHN).[12] At the end of our framework, we use the RegNet to predict the keypoints based on the previous features.

Specifically, MSHNet is based on the convolutional and deconvolutional hourglass module[12] trained with multi-scale loss supervision. The MSHNet achieves the anatomical-aware keypoint regression by matching both the multi-scale keypoint heatmaps and their high-order associations. Both the MSHNet and the RegNet share anatomical-aware supervision (AAS) loss, which is designed to ensure effective multi-scale anatomical feature learning.

There are two reasons for our advanced design based on the vanilla SHN:

1. The convolutional and deconvolutional hourglass stacks are able to capture detailed information for keypoint localization across a wide range of appearances and scales. However, such functionality is highly dependent on a single scale in its multi-scale pyramid and is not able to provide a reliable and stable response for different scales. Therefore, in the development of our MSHNet, we added explicit layer-wise supervisions to all of the deconvolutional layers.

2. The MSHNet hourglass model produces a series of heatmaps, each of which corresponds to the probability of each body keypoint’s position. The heatmaps are supervised against ground-truth ASD keypoint heatmaps, which are

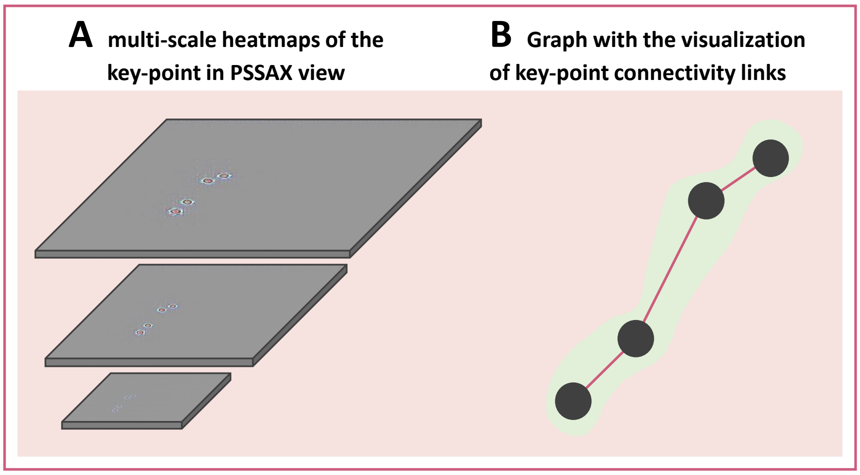

Figure 16. The refinement of keypoint localization in up-sampling in MSHNet works similarly to the attention mechanism used in traditional resolution pyramid scan. (a) the multi-scale heatmaps of a keypoint. (b) our ASD key-point graph with the visualization of keypoint connectivity links, which is sorted by the nearest point as SHN.

usually created using 2D Gaussian blurring, to train the MSHNet. However, the heatmaps acquired during MSHNet research for ASD localization are often non-Gaussian and vary depending on the subject's anatomical structures.

The initial hourglass model[12] has a major flaw in that each keypoint heatmap is estimated individually, ignoring the interaction between the keypoints. In other words, there is no anatomical consistency among detected keypoints.

We add the anatomical-aware loss in between the MSHNet hourglass modules that serve as the aim of intermediate supervision to better catch the adjacency and associations among the ASD keypoints. Therefore, ensuring anatomical consistency in the key-point localization pipeline. The anatomical-aware loss is often used at the end of the pipeline in our RegNet to supervise all keypoint heatmaps at different scales. As a result, the final prediction can be globally consistent.

The RegNet regression matches pairwise consistencies among individual ASD keypoints (first-order consistency) as well as adjacent keypoints (second-order consistency). In contrast to the isolated, uncorrelated individual matches for the final keypoint conclusion, the co-occurrence of a matched pair with high confidence can have a stronger hypothesis. In a joint regression, the RegNet is learned to perform such optimization over all ASD keypoints, all feature scales, and all pairwise correlations.

3.2.1 Multi-scale Hourglass Networks

The multi-scale hourglass networks (MSHNet) were developed with the aim of learning deep features at different scales. At each of the MSHNet's deconvolutional layers, where each layer corresponds to a different scale, we conduct several layer-wise supervision. The box at the bottom of Supplementary Figure 15 depicts the MSHNet framework.

The residual at each deconvolutional layer is calculated using the corresponding down-sampled ground-truth heatmaps in different matching scales (e.g., 1/2, 1/4, 1/8 down-sampling) for multi-scale supervision.

To be more specific, to make the function map dimensions equivalent in order to calculate the residual at the appropriate scales, we resort to the 1×1 convolutional kernel for dimension reduction. We reduce the number of deconvolutional function maps to the number of ASD keypoints in this view. The residual is computed by downsampling the ground-truth keypoint function map to match the corresponding derived keypoint heatmap at each scale.

The multi-scale hourglass networks localize ASD keypoints in analogy to the ‘attention model’ [13] used in the conventional resolution pyramid for image search. The activation areas in the low-resolution heatmaps can be used to guide position refinement in the subsequent high-resolution layers, see Figure. 16.

The multi-scale hourglass networks are trained by L_{MHS}, which is formulated as the L_2 loss from the heatmaps of all keypoints across all scales. We note that this shares some similarities to the multi-scale loss function. [12,14]

To detect the N keypoints, N heatmaps are generated after each convolutional and deconvolutional stack. We note that $N = 3$ in the PSSAX view, and $N = 4$ in SXLAX and A4C views. We note that each of the keypoint labels in each view is recorded as (x, y). The loss at the i-th scale compares the predicted heatmaps of all keypoints against the ground-truth heatmaps at the matching scale:

$$L^i_{MHS} = \frac{1}{N} \sum_{n=1}^{N} \sum_{x,y} ||P_n(x, y) - G_n(x, y)||_2, \tag{1}$$

where $P_n(x, y)$ and $G_n(x, y)$ represent the predicted and the ground-truth confidence maps at the pixel location (x, y) for the n-th keypoint, respectively.

In our dataset, the clinician-labeled coordinates are provided as the keypoint locations. We follow the common practice for ground-truth heatmap generation as in Tompson et al. [15], where the n-th keypoint ground-truth heatmap $G_n(x, y)$ is generated using a 2D Gaussian centered at the keypoint location (x, y), with standard deviation of one pixel.

3.2.2 *Multi-Scale Regression Network*

After the MSHNet with convolutional and deconvolutional stacks, we use a fully-convolutional multi-scale regression network (RegNet) to refine the multi-scale keypoint heatmaps globally and increase anatomical consistency of the estimated keypoints. The multi-scale heatmaps are fed into the RegNet, which matches them to ground-truth keypoints at various scales. The regression network will efficiently integrate heatmaps from all scales to optimize the predicted points in this manner.

The multi-scale regression network jointly optimizes the global ASD anatomical configuration via determining connectivity among ASD keypoints based on the multi-scale features. This can be viewed as an extension to the work of the Convolutional Part Heatmap Regression [16], which only considers keypoint heatmap regression at the scale of the input image. The input image with the keypoint heatmaps can be seen as an attention method and provide a larger resolution. In this case, the multi-scale regression network learns a scale-invariant and attention-based anatomical model, thus providing better performance. In addition, our multi-scale regression network optimizes the anatomical-aware loss, which matches individual keypoints as well as the higher-order association (pairs and triplets of keypoints) in localization ASD keypoints.

3.2.3 *Anatomical-aware Loss*

It has been observed that deeper hourglass stacks lead to better keypoint localization results. [12] As the depth of hourglass stacks increases, gradient vanishing becomes a critical issue in training the network, where intermediate supervision [12,14,17,18] is a common practice to alleviate gradient vanishing.

In order to model the anatomical structure of ASD keypoints, we developed anatomical-aware supervision (AAS) loss function that follows a graph. Specifically, we introduced a graph of ASD keypoints $\mathcal{S}$ (See Figure 16 B for a visualization of the graph.) to define the AAS loss.

Each node $S_n \in \mathcal{S}$ represent a keypoint and its connected keypoints, $n \in \{1, ..., N\}$. The AAS loss at the i-th scale is formally defined as:

$$L^i_{AAS} = \frac{1}{N} \sum_{n=1}^{N} ||P^i_n - G^i_n||_2 + \alpha \sum_{i=1}^{N} ||P^i_{S_n} - G^i_{S_n}||_2. \tag{2}$$

The first term is the multi-scale supervision loss L^i_{MHS} in Eq.1 that represents individual keypoint matching loss. The second term represents the anatomical matching loss, where P_{S_n} and G_{S_n} are the combination of the heatmaps from individual keypoint n and its neighbors in a graph $\mathcal{S}$. Hyperparameter α is a weighing parameter balancing the two terms.

Figure 15 (bottom left) shows a breakdown visualization of how our anatomical-guided AAS loss is calculated in traversing the keypoints and their relationships according to $\mathcal{S}$. The top row in the sub-figure shows the intermediate loss defined on individual keypoints as used in.[12,14] The bottom row shows our anatomical-aware supervision loss defined for a set of connected keypoints.

We apply the AAS loss for two procedures:

1. In the middle of the MSHNet stacks as a means of intermediate overseeing to ensure anatomical accuracy and localizing keypoints;

2. In the RegNet to explore a globally consistent keypoint configuration.

4 Training Details

4.1 Platform

Our framework was implemented using the PyTorch deep learning toolbox.[19] Training of our deep key-point stadiometry was performed on four NVIDIA V100 GPUs, which took about 6 hours. The keypoint localization of an echocardiogram in the test stage only takes 0·2 seconds with a single GPU.

4.2 Hyperparameter Search

All model architectures were tuned with Bayesian Optimization using the HyperOpt library[20] and a Tree-structured Parzen Estimator[21] in the validation set with 30 subjects (20 transcatheter closures and 10 surgical closures). Each architecture was trained with 30 evaluations through the same hyperparameter search space defined in Supplementary Table 3.

Table 3. Selection range of the hyperparameters

Hyperparameters	Range	Stride
α	0-2	0·1
Dropout Probability	0·1-0·5	0·05
Learning Rate	0·0001-0·001	0·0001
L2-regularization	0·001-0·01	0·01
ADAM optimizer (1-100 epochs)	0·5-0·9	0·1
ADAM optimizer (100-200 epochs)	0·9-0·99	0·01

Hyperparameter optimization was set up to maximize peak training PCK. Testing performance was not computed until reporting the final results. Best performing architectures were then tested on the testing set. We note that both DKS and black-box models are tuned using the same protocol.

4.3 Training Setup

All models were trained to 200 epochs, well beyond convergence which typically occurred at 50 to 100 epochs. Instead, the “black-box" model is usually saturated after 50 epochs. The batch size is set to two in all of our “black-box" and DKS models.

4.4 Class Balancing in “Black-box" Model

Due to the uneven distribution of outcomes, gradient descent step weights were weighted so that all outcomes would receive an even expected step accumulation from both positive and negative examples. Using statistics from training label occurrences, this was enforced, giving negative labels a weighting factor of and further multiplying steps by a class normalization factor so that no single class dominates the optimization weighting. This combination of weighting consistently resulted in relatively balanced performance between classes.

We note that the DKS model does not directly predict the classes and does not need this operation.

5 Evaluation Metrics

The first objective of our study was the Percentage of Correct Keypoints (PCK) metric [15] of key-point localization in three views. Specifically, our PCK is calculated as the percentage of disparities between the detected keypoints w.r.t. the ground-truth after normalization against a fraction of the measuring scale length.

Then, we evaluate the accuracy, F1 score, sensitivity, and specificity for the classification of transcatheter or surgical closure suggestions. We note that the DKS model does not have a threshold for binary classification as the "black-bock" model. Therefore, the receiver operating characteristic is not applicable here.

The widely accepted binary classification metrics of F1 score, accuracy, sensitivity (i.e., recall), and specificity can be formulated as:

$$F1 = \frac{TP}{TP + \frac{TP+FN}{2}}, \tag{3}$$

$$accuracy = \frac{TP + TN}{TP + TN + FP + FN}, \tag{4}$$

$$sensitivity = \frac{TP}{TP + FN}, \tag{5}$$

$$specificity = \frac{TN}{TN + FP}, \tag{6}$$

where TP, TN, FP, and FN indicate true positive, true negative, false positive, and false negative, respectively. We note that the positive and negative correspond to the transcatheter and surgical, respectively.

The suggested ASD occluder size is also proposed for the transcatheter closure cases. We adopt the metrics of mean absolute error (MAE) and the quadratic weighted kappa (QWK)[4] that are used in the ordinal classification task.[22,23] Specifically, the QWK is formulated as:

$$k = 1 - \frac{\sum_{i,j} \mathbf{W}_{i,j}\mathbf{O}_{i,j}}{\sum_{i,j} \mathbf{W}_{i,j}\mathbf{E}_{i,j}} \tag{7}$$

to measure the level of disagreement between two raters ($\mathcal{A}$ and $\mathcal{B}$). Here, the $\mathcal{A}$ is the prediction of our DHS, and $\mathcal{B}$ is the ground truth. The $\mathbf{W}$ is a $N \times N$ matrix where $\mathbf{W}_{i,j}$ denotes the cost associated with misclassifying label i as label j. In QWK, $\mathbf{W}_{i,j} = (i-j)^2$. $\mathbf{O}_{i,j}$ counts the number of images that received a rating i by $\mathcal{A}$ and a rating j by $\mathcal{B}$. The quadratic calculation is one possible choice, and one can plug in other distance metrics into the kappa calculation. The matrix of expected ratings $\mathbf{E}$, is calculated, assuming that there is no correlation between rating scores. As a result, k is a scalar in [-1,1], and $k = 1$ indicates the two raters are in total agreement, whereas $k < 0$ means the classifier performs worse than the random choice.

MAE and QWK can punish the misclassification proportional to the distance between the predicted label of the network and the ground truth label.

[4] https://www.kaggle.com/c/diabetic-retinopathy-detection\#evaluation

6 Failure Analysis

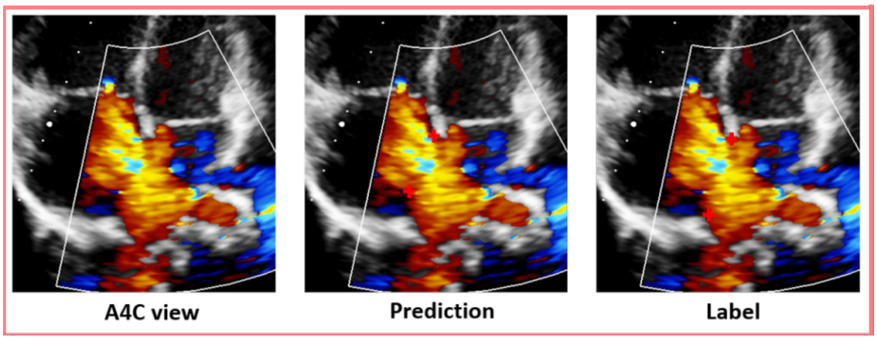

Figure 17. The Failure example of our DKS model. The surgical closure sample is predicted to transcatheter closure. keypoints are indicated with red cross.

In Figure 17, we show a failure example of our DKS model. The keypoints for assessing the length of the defect in the A4C view, and the predicted keypoint (bottom left point) tend to have a shorter defect length than the manual label, which leads to a surgical closure sample being misclassified as transcatheter closure. We can see that Doppler imaging can cover some parts of the anatomical structure and make the keypoint localization ambiguous. The strong anatomical knowledge of experienced clinicians can be necessary to correct these cases. In addition, the retest or the other imaging modalities may be helpful in providing more complementary information for the final decision making.

References

[1] Campbell M. Natural history of atrial septal defect. Heart. 1970;32(6):820–826.

[2] Villablanca PA, Briston DA, Rodés-Cabau J, Briceno DF, Rao G, Aljoudi M, et al. Treatment options for the closure of secundum atrial septal defects: a systematic review and meta-analysis. International journal of cardiology. 2017;241:149–155.

[3] Jalal Z, Hascoet S, Baruteau AE, Iriart X, Kreitmann B, Boudjemline Y, et al. Long-term complications after transcatheter atrial septal defect closure: a review of the medical literature. Canadian Journal of Cardiology. 2016;32(11):1315–e11.

[4] Bartel T, Konorza T, Barbieri V, Erbel R, Pachinger O, Müller S. Single-plane balloon sizing of atrial septal defects with intracardiac echocardiography: an advantageous alternative to fluoroscopy. Journal of the American Society of Echocardiography. 2008;21(6):737–740.

[5] Gupta SK, Sivasankaran S, Bijulal S, Tharakan JM, Harikrishnan S, Ajit K. Trans-catheter closure of atrial septal defect: balloon sizing or no balloon sizing–single centre experience. Annals of pediatric cardiology. 2011;4(1):28.

[6] Alqahtani F, Bhirud A, Aljohani S, Mills J, Kawsara A, Runkana A, et al. Intracardiac versus transesophageal echocardiography to guide transcatheter closure of interatrial communications: nationwide trend and comparative analysis. Journal of interventional cardiology. 2017;30(3):234–241.

[7] Adhikari CM, Bogati A, Prajapati D, Dhungel S, Najmy S, Acharya K, et al. Atrial Septal Defect Size and Rims on Transesophageal Echocardiogram. Maedica. 2019;14(2):81.

[8] Committee CHD, Association CPP, Association CMD, et al. Expert consensus for the interventional treatment of pediatric congenital heart disease. Zhonghua er ke za zhi= Chinese journal of pediatrics. 2015;53(1):17.

[9] Anavekar NS, Oh JK. Doppler echocardiography: a contemporary review. Journal of cardiology. 2009;54(3):347–358.

[10] Wang J, Liu X, Wang F, Zheng L, Gao F, Zhang H, et al. Automated interpretation of congenital heart disease from multi-view echocardiograms. Medical Image Analysis. 2021;69:101942.

[11] Howard AG, Zhu M, Chen B, Kalenichenko D, Wang W, Weyand T, et al. Mobilenets: Efficient convolutional neural networks for mobile vision applications. arXiv preprint arXiv:170404861. 2017.

[12] Newell A, Yang K, Deng J. Stacked hourglass networks for human pose estimation. In: European conference on computer vision. Springer; 2016. p. 483–499.

[13] Zhao B, Wu X, Feng J, Peng Q, Yan S. Diversified visual attention networks for fine-grained object classification. IEEE Transactions on Multimedia. 2017;19(6):1245–1256.

[14] Wei SE, Ramakrishna V, Kanade T, Sheikh Y. Convolutional pose machines. In: Proceedings of the IEEE conference on Computer Vision and Pattern Recognition; 2016. p. 4724–4732.

[15] Tompson J, Goroshin R, Jain A, LeCun Y, Bregler C. Efficient object localization using convolutional networks. In: Proceedings of the IEEE conference on computer vision and pattern recognition; 2015. p. 648–656.

[16] Bulat A, Tzimiropoulos G. Human pose estimation via convolutional part heatmap regression. In: European Conference on Computer Vision. Springer; 2016. p. 717–732.

[17] Chu X, Ouyang W, Li H, Wang X. Structured feature learning for pose estimation. In: Proceedings of the IEEE Conference on Computer Vision and Pattern Recognition; 2016. p. 4715–4723.

[18] Yang W, Li S, Ouyang W, Li H, Wang X. Learning feature pyramids for human pose estimation. In: proceedings of the IEEE international conference on computer vision; 2017. p. 1281–1290.

[19] Paszke A, Gross S, Massa F, Lerer A, Bradbury J, Chanan G, et al. Pytorch: An imperative style, high-performance deep learning library. arXiv preprint arXiv:191201703. 2019.

[20] Bergstra J, Yamins D, Cox D. Making a science of model search: Hyperparameter optimization in hundreds of dimensions for vision architectures. In: International conference on machine learning. PMLR; 2013. p. 115–123.

[21] Bergstra J, Bardenet R, Bengio Y, Kégl B. Algorithms for hyper-parameter optimization. In: 25th annual conference on neural information processing systems (NIPS 2011). vol. 24. Neural Information Processing Systems Foundation; 2011. .

[22] Cohen J. Weighted kappa: Nominal scale agreement provision for scaled disagreement or partial credit. Psychological bulletin. 1968;70(4):213.

[23] Liu X, Han X, Qiao Y, Ge Y, Li S, Lu J. Unimodal-uniform constrained wasserstein training for medical diagnosis. In: Proceedings of the IEEE/CVF International Conference on Computer Vision Workshops; 2019. p. 0–0.

List of Figures

1 Illustration of the ASD (A), and its repair plan: transcatheter closure (B) and surgical closure (C). . . 3

2 Illustration of the ASD occulder in transcatheter closure. (take the Amplatzer™ Septal Occluder as an example) 4

3 Illustration of the key-frame selection. We use A4C view as an example. 7

4 Illustration of the measuring scale. 8

5 Illustration of a subject with 2D and Doppler echocardiograms in three views. PSSAX: short for PSSAX of the aorta; SXLAX: short for SXLAX of two atria 9

6 The labeling of 2D echocardiogram in the view of the view of PSSAX of the aorta 9

7 The labeling of Doppler echocardiogram in the view of PSSAX of the aorta 9

8 The labeling of 2D echocardiogram in the view of SXLAX of two atria. 10

9 The labeling of Doppler echocardiogram in the view of SXLAX of two atria. 10

10 The labeling of 2D echocardiogram in the view of A4C. 11

11 The labeling of Doppler echocardiogram in the view of A4C. 11

12 Illustration of the two-dimensional label of the keypoint. 12

13 Prepossessing of the echocardiograms. 12

14 The network structure of the “black-box" classification model. PSSAX: short for PSSAX of the aorta; SXLAX: short for SXLAX of two atria 13

15 The detailed network structure for key-point localization. MSHNet: multi-scale hourglass networks, RegNet: regression network. 14

16 The refinement of keypoint localization in up-sampling in MSHNet works similarly to the attention mechanism used in traditional resolution pyramid scan. (a) the multi-scale heatmaps of a keypoint. (b) our ASD key-point graph with the visualization of keypoint connectivity links, which is sorted by the nearest point as SHN. 15

17 The Failure example of our DKS model. The surgical closure sample is predicted to transcatheter closure. keypoints are indicated with red cross. 20

List of Tables

1 The common information of occluder and the distribution of occluder size in transcatheter subset. (take the Amplatzer™ Septal Occluder as an example) 6

2 The detailed structure of our "black-box" classification model. 13

3 Selection range of the hyperparameters 18
