# Supplementary material for: Assessment of Transcatheter or Surgical Closure of Atrial Septal Defect using Interpretable Deep Keypoint Stadiometry
Source: Research (Wash D C). 2022 Oct 21;2022:9790653. doi: 10.34133/2022/9790653 (PMC9620637; doi:10.34133/2022/9790653)
Supplement: Supplementary Materials — The supplementary incorporates the task background, data collection, model details, evaluation metrics, and failure case analysis. Figure S1: the accuracy concerning the number of training epochs for the “black-box” model and deep keypoint stadiometry model. Figure S2: comparison of the occluder size prediction with MAE (the smaller, the better) and QWK (the larger, the better) metrics. Supplementary Table 1: the statistics of the clinical characteristics of collected ASD patients. [file 9790653.f1.zip › Supplementary Table 1.docx]

**Supplementary Table 1.** **The statistics of the clinical characteristics of collected ASD patients. Data are n, n (%), or mean (±SD). Estimated based on the average of clinicians’ labelling.** * We adopt the one-tailed paired t-test to report the p-value use within-center data as reference.

| Dataset | First evaluation dataset | | Second evaluation dataset | | | |
| --- | --- | --- | --- | --- | --- | --- |
| Closure Method | Transcatheter | Surgical | Transcatheter | p-value* | Surgical | p-value* |
| Number of participants | 250 (55.6%) | 200 (44.4%) | 110 (85.3%) | - | 19 (14.7%) | - |
| Gender (male/female) | 107/143 | 77/123 | 48/62 | - | 7/12 | - |
| Age (years) | 5.29 ±3.39 | 2.85 ±2.05 | 6.36 ±3.38 | - | 3.52 ±2.45 | - |
| PSSAX of the aorta View: | | | | | | |
| Defect diameter (mm) | 10.1 ±2.2 | 19.3 ±2.4 | 9.6 ±1.7 | 0.474 | 18.5 ±3.1 | 0.352 |
| To Atrial posterior wall (mm) | 8.2 ±1.4 | 4.8 ±1.1 | 9.5 ±1.4 | 0.35 | 5.9 ±1.4 | 0.481 |
| SXLAX of two atria View: | | | | | | |
| Defect diameter (mm) | 10.3 ±2.5 | 19.4 ±2.7 | 11.4 ±2.8 | 0.295 | 17.8 ±1.9 | 0.258 |
| To superior vena cava (mm) | 8.5 ±1.1 | 5.4 ±0.9 | 8.3 ±2.1 | 0.517 | 6.6 ±1.3 | 0.361 |
| To inferior vena cava (mm) | 9.3 ±1.2 | 5.1 ±1.5 | 9.5 ±1.8 | 0.386 | 7.2 ±1.7 | 0.329 |
| A4C View: | | | | | | |
| Defect diameter (mm) | 8.5 ±2.7 | 18.8 ±3.2 | 9.0 ±3.3 | 0.352 | 17.7 ±2.8 | 0.34 |
| To atrial roof (mm) | 9.4 ±1.7 | 7.2 ±1.6 | 9.6 ±1.5 | 0.431 | 7.1 ±2.1 | 0.525 |
| To mitral annulus (mm) | 10.5 ±1.3 | 6.9 ±1.4 | 9.7 ±1.6 | 0.387 | 6.6 ±1.2 | 0.426 |
| Atrial septum length (mm) | 30.3 ±3.2 | 30.2 ±3.7 | 28.6 ±2.9 | 0.323 | 29.0 ±2.5 | 0.327 |
