# Supplementary figures and images for: Assessment of Transcatheter or Surgical Closure of Atrial Septal Defect using Interpretable Deep Keypoint Stadiometry
Source: Research (Wash D C). 2022 Oct 21;2022:9790653. doi: 10.34133/2022/9790653 (PMC9620637; doi:10.34133/2022/9790653)

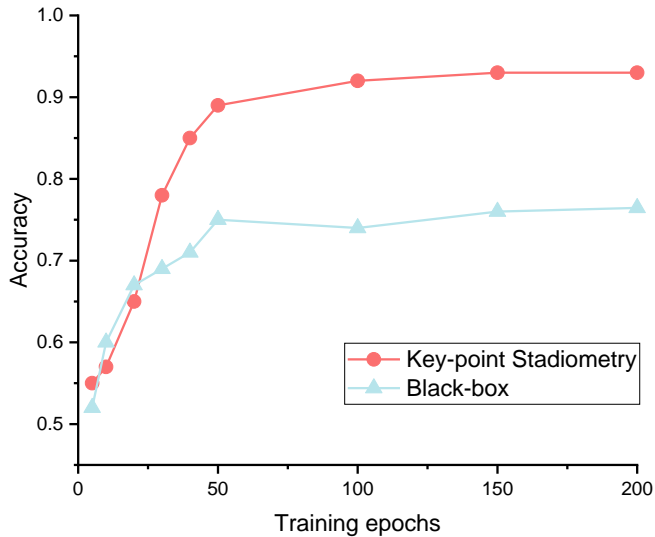

Supplement: Supplementary Materials — The supplementary incorporates the task background, data collection, model details, evaluation metrics, and failure case analysis. Figure S1: the accuracy concerning the number of training epochs for the “black-box” model and deep keypoint stadiometry model. Figure S2: comparison of the occluder size prediction with MAE (the smaller, the better) and QWK (the larger, the better) metrics. Supplementary Table 1: the statistics of the clinical characteristics of collected ASD patients. [file 9790653.f1.zip › Figure S1.pdf]

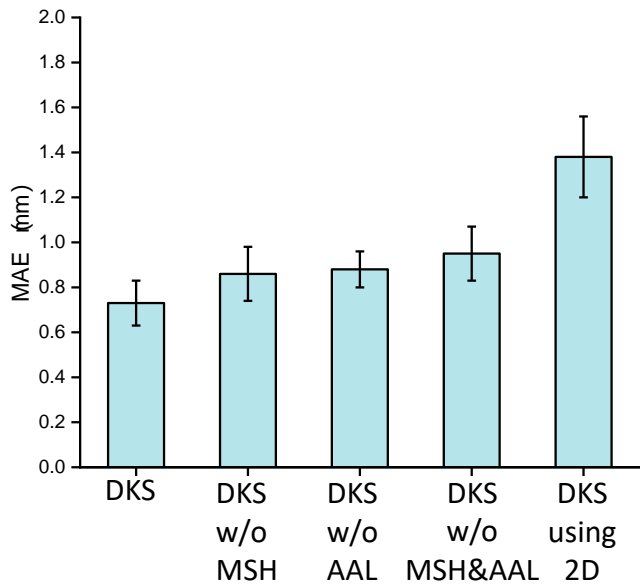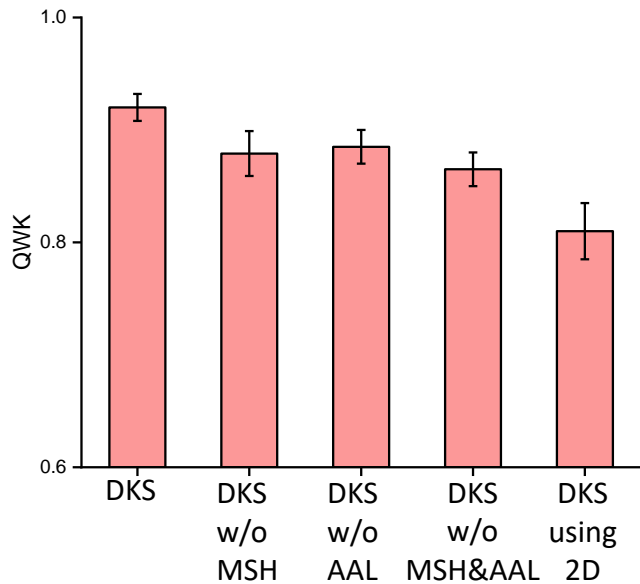

Supplement: Supplementary Materials — The supplementary incorporates the task background, data collection, model details, evaluation metrics, and failure case analysis. Figure S1: the accuracy concerning the number of training epochs for the “black-box” model and deep keypoint stadiometry model. Figure S2: comparison of the occluder size prediction with MAE (the smaller, the better) and QWK (the larger, the better) metrics. Supplementary Table 1: the statistics of the clinical characteristics of collected ASD patients. [file 9790653.f1.zip › Figure S2.pdf]

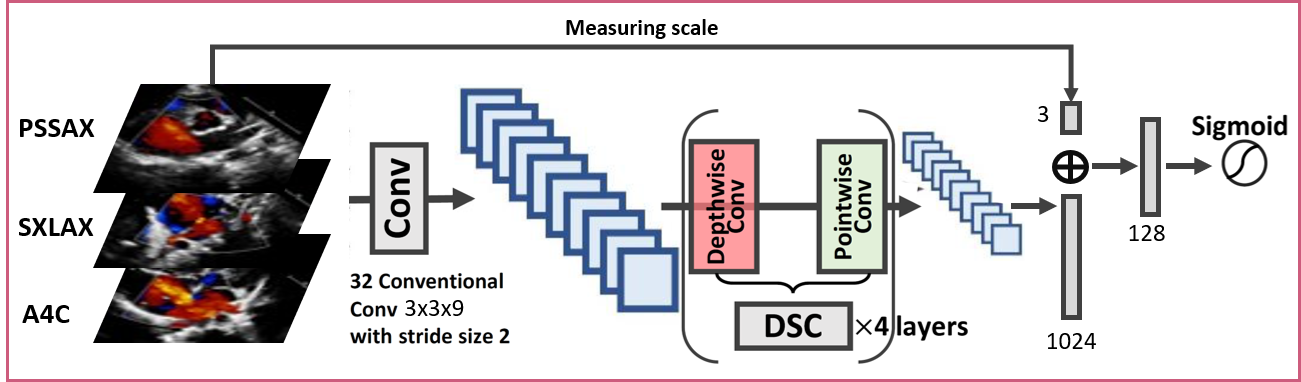

Supplement: Supplementary Materials — The supplementary incorporates the task background, data collection, model details, evaluation metrics, and failure case analysis. Figure S1: the accuracy concerning the number of training epochs for the “black-box” model and deep keypoint stadiometry model. Figure S2: comparison of the occluder size prediction with MAE (the smaller, the better) and QWK (the larger, the better) metrics. Supplementary Table 1: the statistics of the clinical characteristics of collected ASD patients. [file 9790653.f1.zip › Research(supplymentary)/images/black.png]

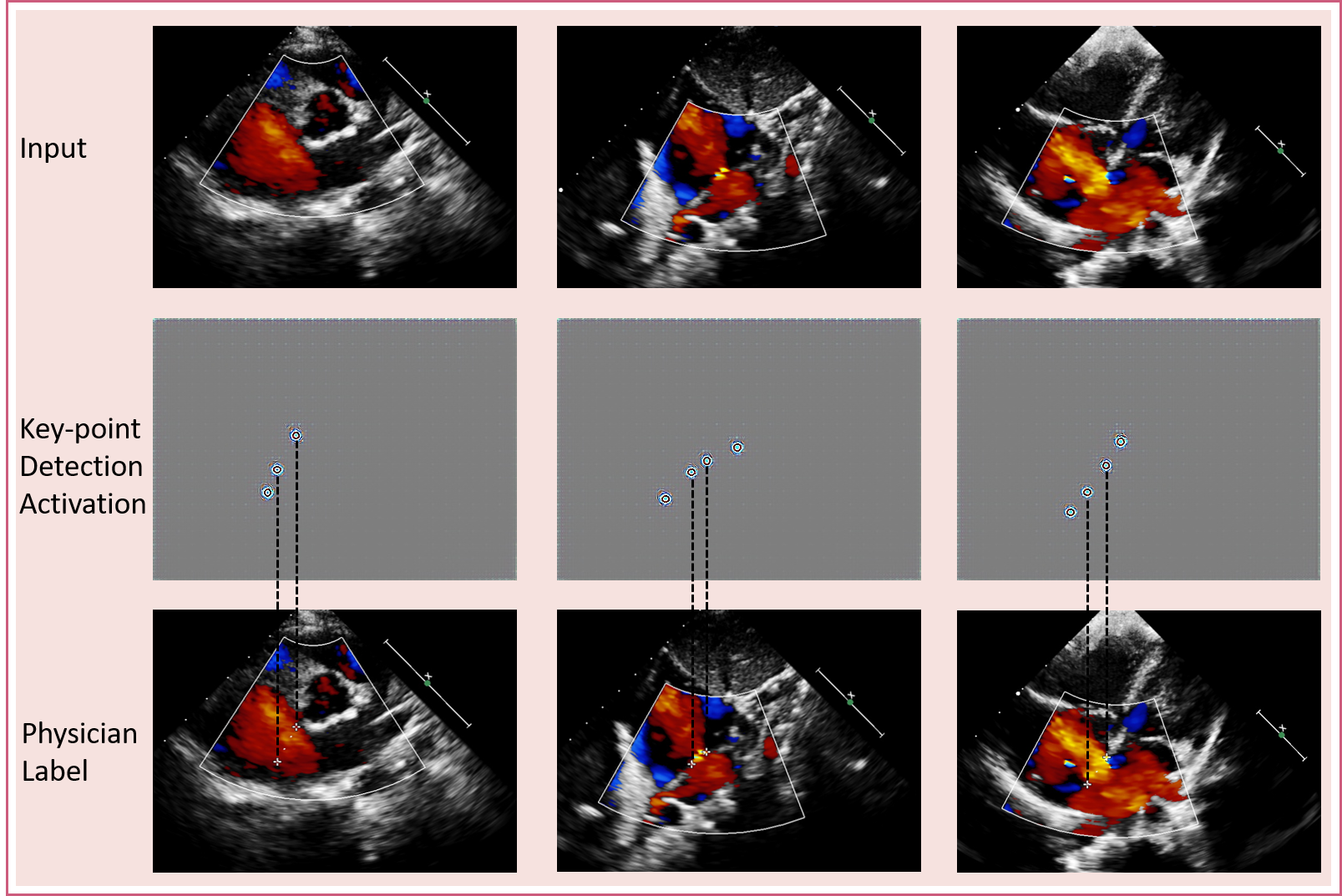

Supplement: Supplementary Materials — The supplementary incorporates the task background, data collection, model details, evaluation metrics, and failure case analysis. Figure S1: the accuracy concerning the number of training epochs for the “black-box” model and deep keypoint stadiometry model. Figure S2: comparison of the occluder size prediction with MAE (the smaller, the better) and QWK (the larger, the better) metrics. Supplementary Table 1: the statistics of the clinical characteristics of collected ASD patients. [file 9790653.f1.zip › Research(supplymentary)/images/explancet1.png]

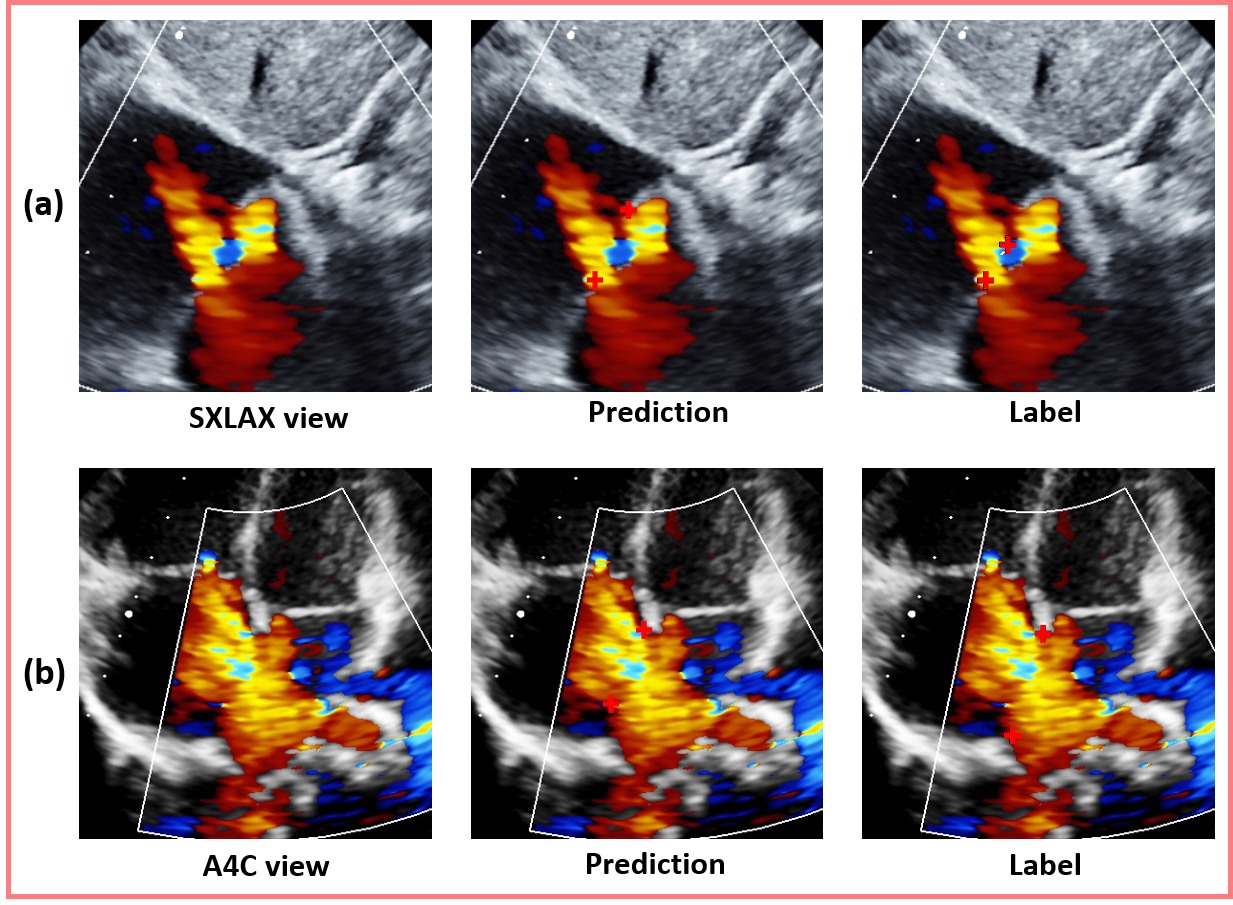

Supplement: Supplementary Materials — The supplementary incorporates the task background, data collection, model details, evaluation metrics, and failure case analysis. Figure S1: the accuracy concerning the number of training epochs for the “black-box” model and deep keypoint stadiometry model. Figure S2: comparison of the occluder size prediction with MAE (the smaller, the better) and QWK (the larger, the better) metrics. Supplementary Table 1: the statistics of the clinical characteristics of collected ASD patients. [file 9790653.f1.zip › Research(supplymentary)/images/falure.png]

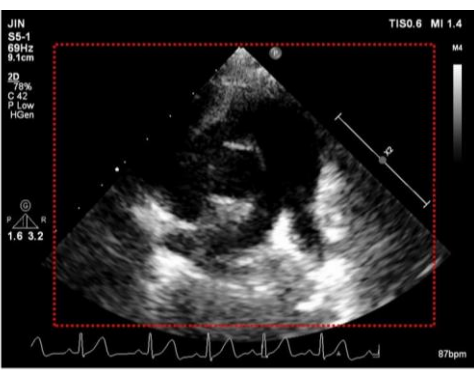

ROI crop

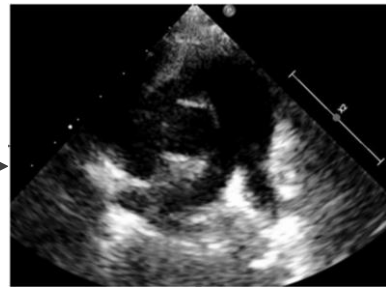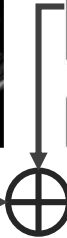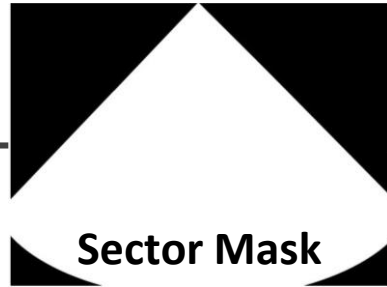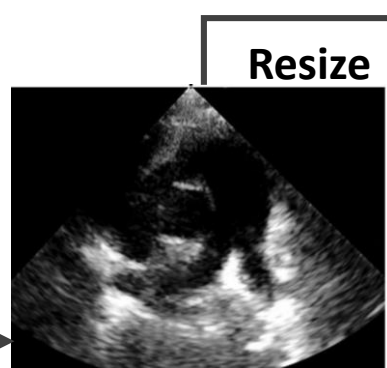

Resize

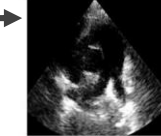

$128 \times 128$

⋮

Supplement: Supplementary Materials — The supplementary incorporates the task background, data collection, model details, evaluation metrics, and failure case analysis. Figure S1: the accuracy concerning the number of training epochs for the “black-box” model and deep keypoint stadiometry model. Figure S2: comparison of the occluder size prediction with MAE (the smaller, the better) and QWK (the larger, the better) metrics. Supplementary Table 1: the statistics of the clinical characteristics of collected ASD patients. [file 9790653.f1.zip › Research(supplymentary)/images/Fig 2.pdf]

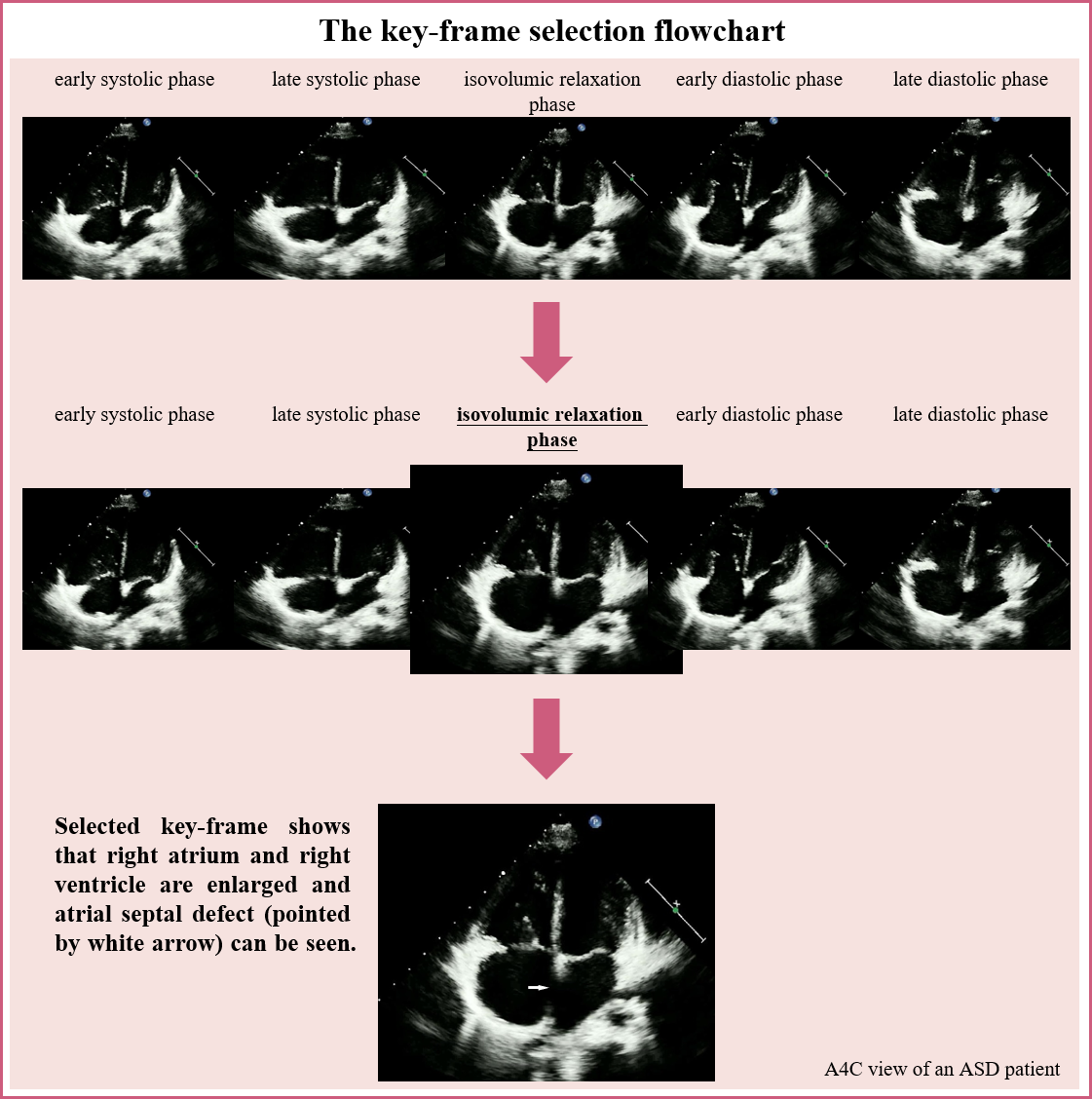

Supplement: Supplementary Materials — The supplementary incorporates the task background, data collection, model details, evaluation metrics, and failure case analysis. Figure S1: the accuracy concerning the number of training epochs for the “black-box” model and deep keypoint stadiometry model. Figure S2: comparison of the occluder size prediction with MAE (the smaller, the better) and QWK (the larger, the better) metrics. Supplementary Table 1: the statistics of the clinical characteristics of collected ASD patients. [file 9790653.f1.zip › Research(supplymentary)/images/figflow.png]

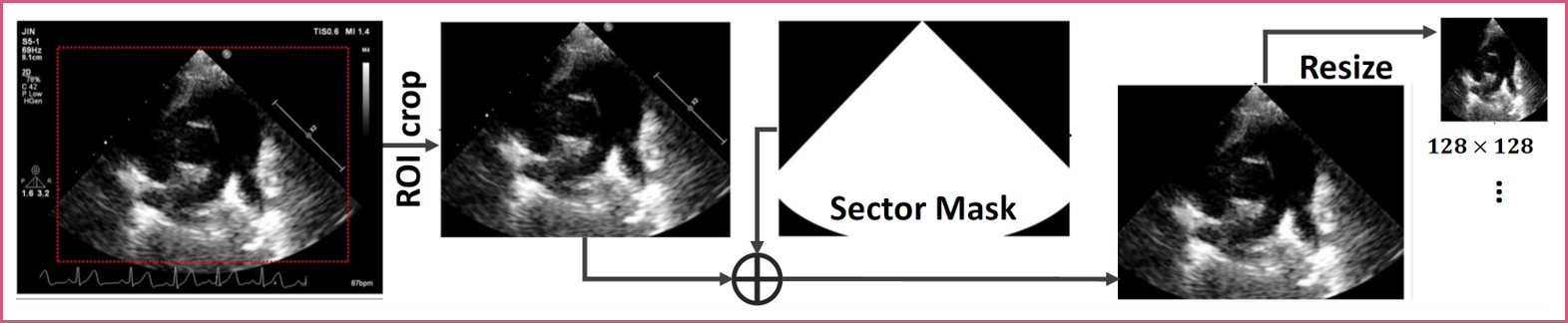

Supplement: Supplementary Materials — The supplementary incorporates the task background, data collection, model details, evaluation metrics, and failure case analysis. Figure S1: the accuracy concerning the number of training epochs for the “black-box” model and deep keypoint stadiometry model. Figure S2: comparison of the occluder size prediction with MAE (the smaller, the better) and QWK (the larger, the better) metrics. Supplementary Table 1: the statistics of the clinical characteristics of collected ASD patients. [file 9790653.f1.zip › Research(supplymentary)/images/figpre.png]

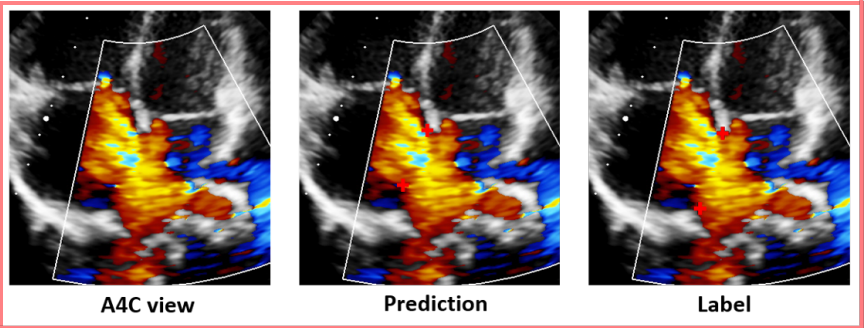

Supplement: Supplementary Materials — The supplementary incorporates the task background, data collection, model details, evaluation metrics, and failure case analysis. Figure S1: the accuracy concerning the number of training epochs for the “black-box” model and deep keypoint stadiometry model. Figure S2: comparison of the occluder size prediction with MAE (the smaller, the better) and QWK (the larger, the better) metrics. Supplementary Table 1: the statistics of the clinical characteristics of collected ASD patients. [file 9790653.f1.zip › Research(supplymentary)/images/Figure17.png]

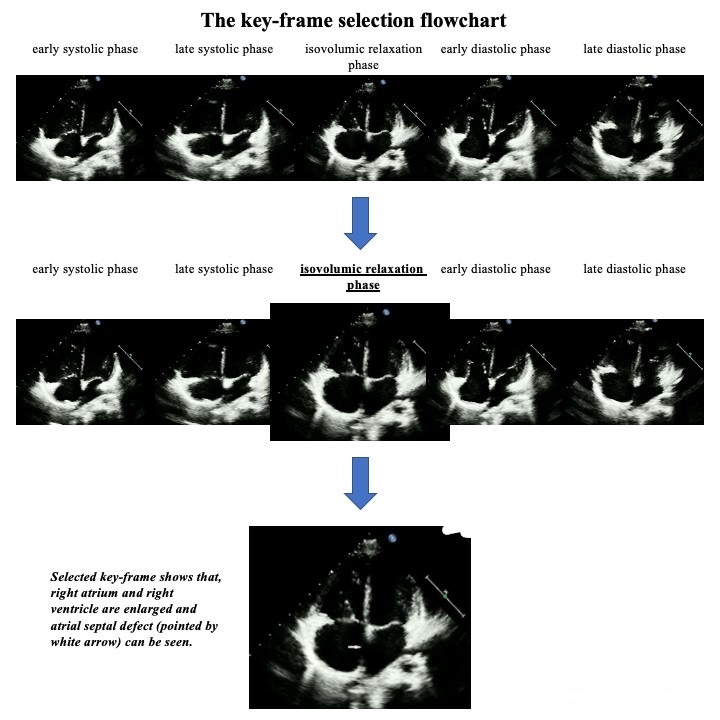

Supplement: Supplementary Materials — The supplementary incorporates the task background, data collection, model details, evaluation metrics, and failure case analysis. Figure S1: the accuracy concerning the number of training epochs for the “black-box” model and deep keypoint stadiometry model. Figure S2: comparison of the occluder size prediction with MAE (the smaller, the better) and QWK (the larger, the better) metrics. Supplementary Table 1: the statistics of the clinical characteristics of collected ASD patients. [file 9790653.f1.zip › Research(supplymentary)/images/InkedInkedfigflow.jpg]

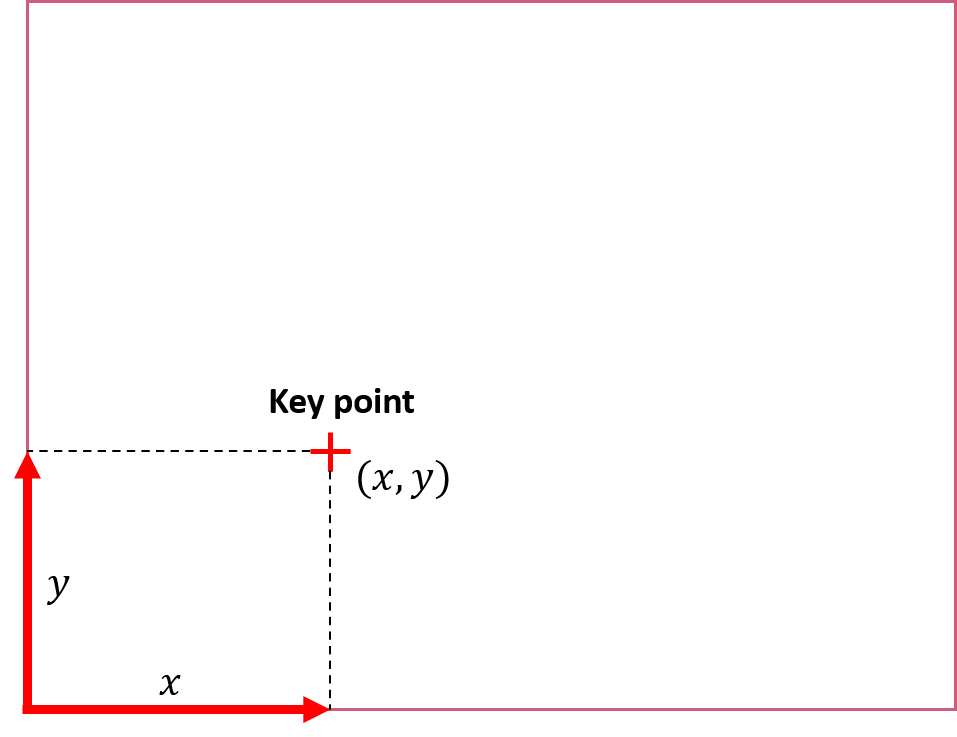

Supplement: Supplementary Materials — The supplementary incorporates the task background, data collection, model details, evaluation metrics, and failure case analysis. Figure S1: the accuracy concerning the number of training epochs for the “black-box” model and deep keypoint stadiometry model. Figure S2: comparison of the occluder size prediction with MAE (the smaller, the better) and QWK (the larger, the better) metrics. Supplementary Table 1: the statistics of the clinical characteristics of collected ASD patients. [file 9790653.f1.zip › Research(supplymentary)/images/lancetkey.png]

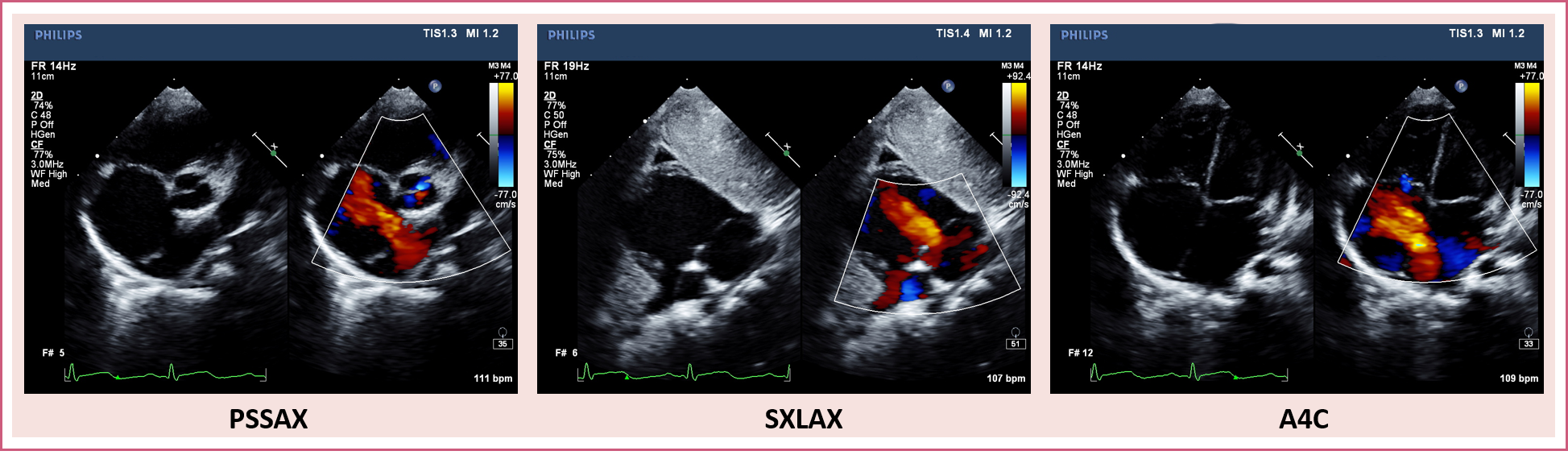

Supplement: Supplementary Materials — The supplementary incorporates the task background, data collection, model details, evaluation metrics, and failure case analysis. Figure S1: the accuracy concerning the number of training epochs for the “black-box” model and deep keypoint stadiometry model. Figure S2: comparison of the occluder size prediction with MAE (the smaller, the better) and QWK (the larger, the better) metrics. Supplementary Table 1: the statistics of the clinical characteristics of collected ASD patients. [file 9790653.f1.zip › Research(supplymentary)/images/lancetsample.png]

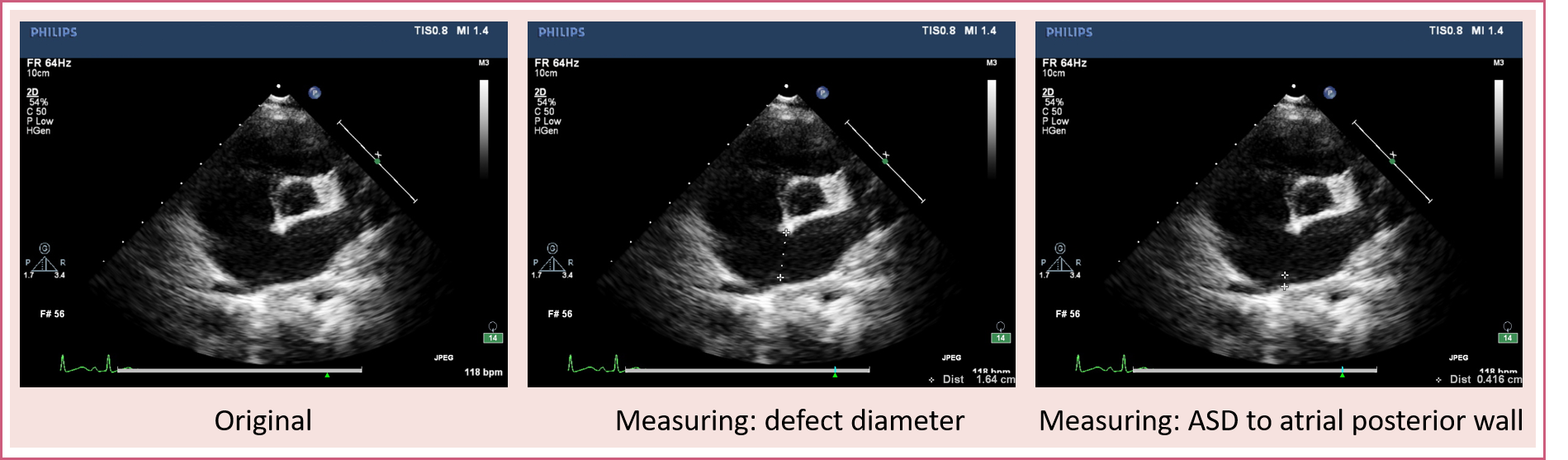

Supplement: Supplementary Materials — The supplementary incorporates the task background, data collection, model details, evaluation metrics, and failure case analysis. Figure S1: the accuracy concerning the number of training epochs for the “black-box” model and deep keypoint stadiometry model. Figure S2: comparison of the occluder size prediction with MAE (the smaller, the better) and QWK (the larger, the better) metrics. Supplementary Table 1: the statistics of the clinical characteristics of collected ASD patients. [file 9790653.f1.zip › Research(supplymentary)/images/lancetsample111a.png]

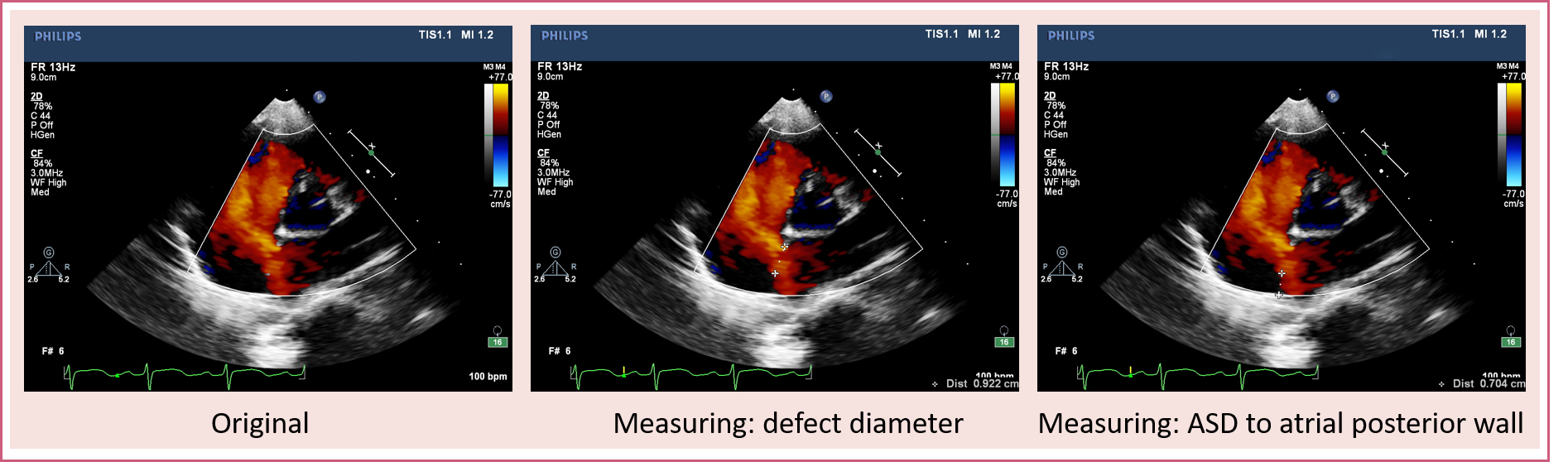

Supplement: Supplementary Materials — The supplementary incorporates the task background, data collection, model details, evaluation metrics, and failure case analysis. Figure S1: the accuracy concerning the number of training epochs for the “black-box” model and deep keypoint stadiometry model. Figure S2: comparison of the occluder size prediction with MAE (the smaller, the better) and QWK (the larger, the better) metrics. Supplementary Table 1: the statistics of the clinical characteristics of collected ASD patients. [file 9790653.f1.zip › Research(supplymentary)/images/lancetsample111b.png]

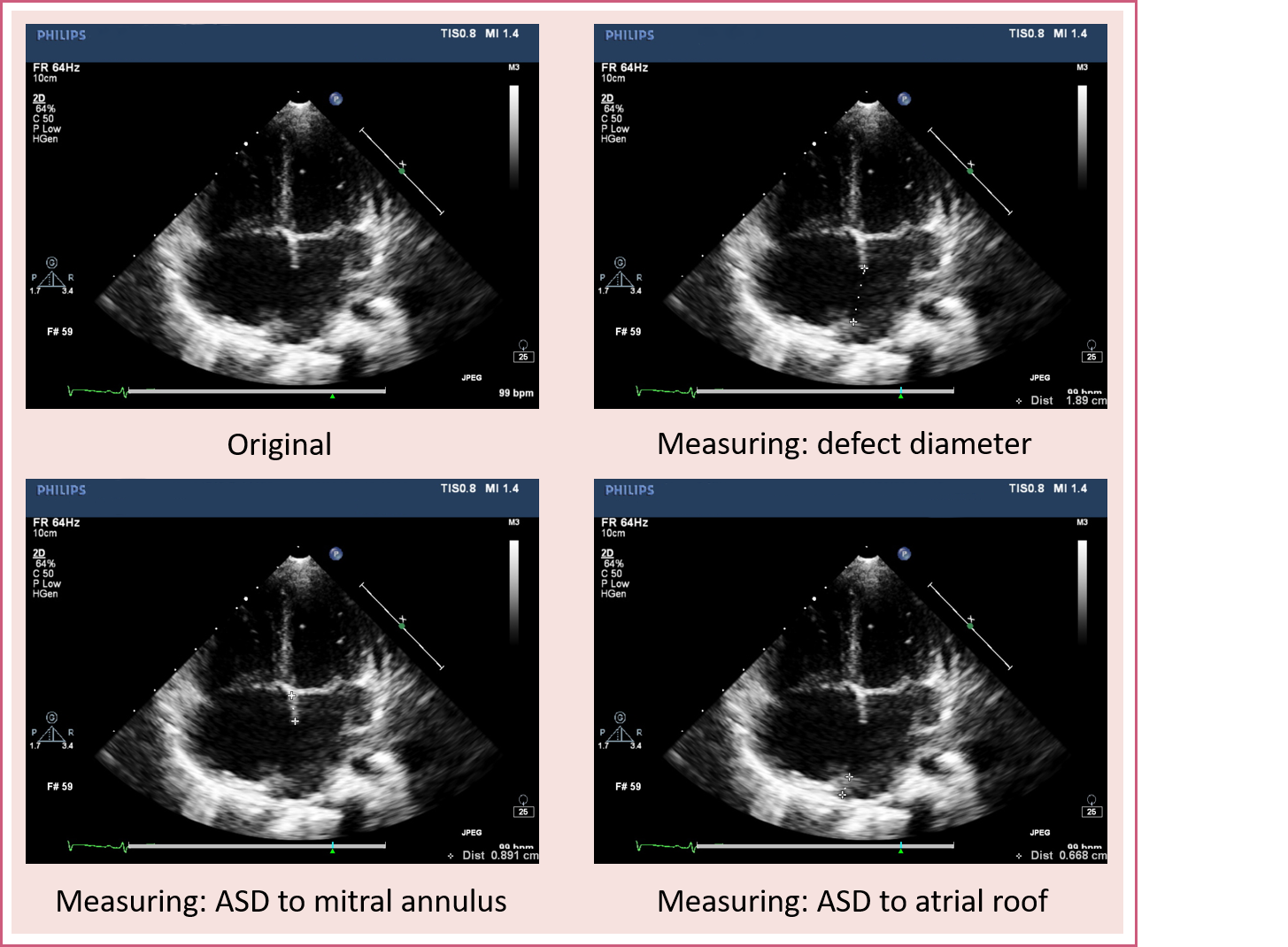

Supplement: Supplementary Materials — The supplementary incorporates the task background, data collection, model details, evaluation metrics, and failure case analysis. Figure S1: the accuracy concerning the number of training epochs for the “black-box” model and deep keypoint stadiometry model. Figure S2: comparison of the occluder size prediction with MAE (the smaller, the better) and QWK (the larger, the better) metrics. Supplementary Table 1: the statistics of the clinical characteristics of collected ASD patients. [file 9790653.f1.zip › Research(supplymentary)/images/lancetsample222a.png]

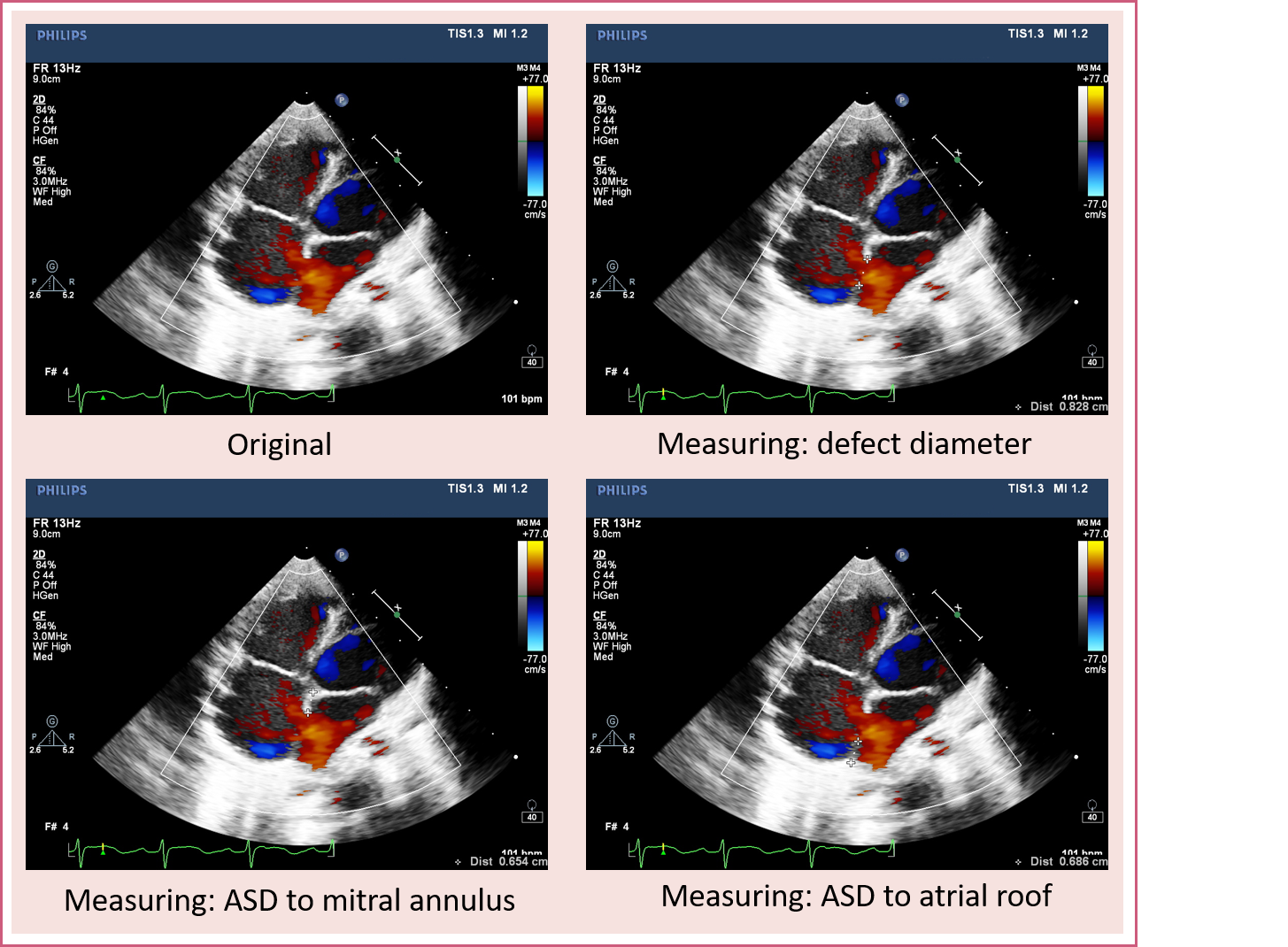

Supplement: Supplementary Materials — The supplementary incorporates the task background, data collection, model details, evaluation metrics, and failure case analysis. Figure S1: the accuracy concerning the number of training epochs for the “black-box” model and deep keypoint stadiometry model. Figure S2: comparison of the occluder size prediction with MAE (the smaller, the better) and QWK (the larger, the better) metrics. Supplementary Table 1: the statistics of the clinical characteristics of collected ASD patients. [file 9790653.f1.zip › Research(supplymentary)/images/lancetsample222b.png]

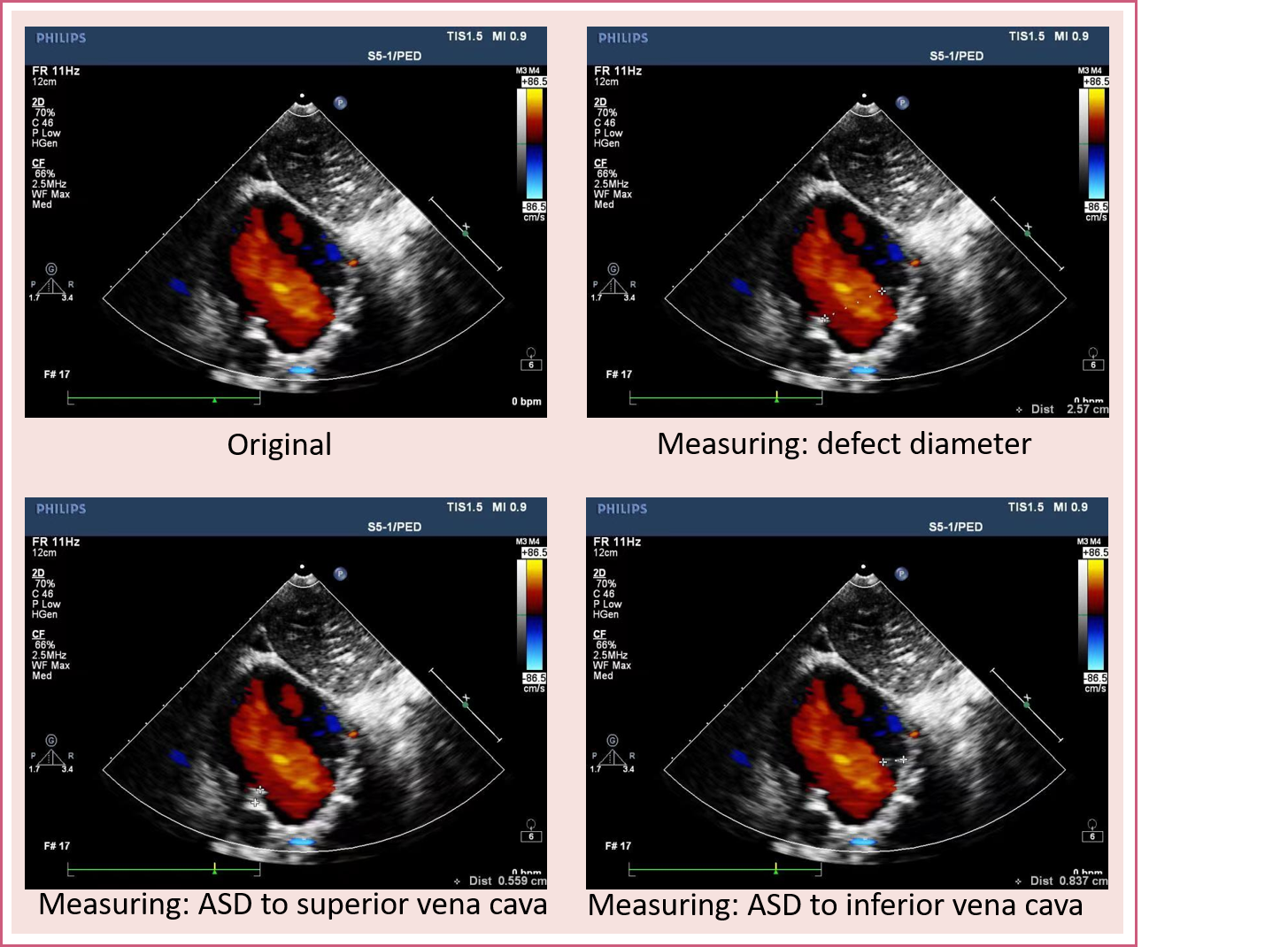

Supplement: Supplementary Materials — The supplementary incorporates the task background, data collection, model details, evaluation metrics, and failure case analysis. Figure S1: the accuracy concerning the number of training epochs for the “black-box” model and deep keypoint stadiometry model. Figure S2: comparison of the occluder size prediction with MAE (the smaller, the better) and QWK (the larger, the better) metrics. Supplementary Table 1: the statistics of the clinical characteristics of collected ASD patients. [file 9790653.f1.zip › Research(supplymentary)/images/lancetsample333.png]

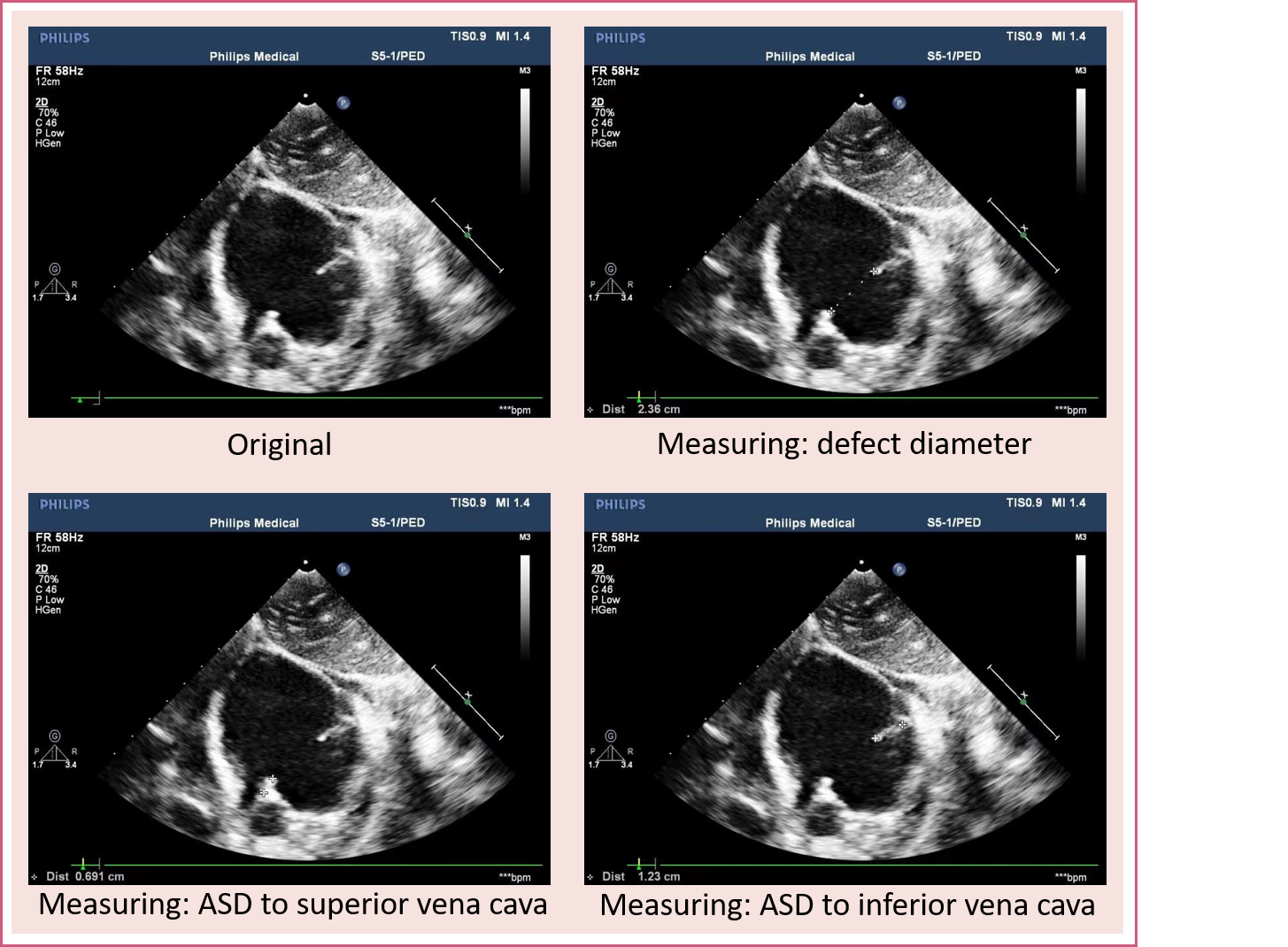

Supplement: Supplementary Materials — The supplementary incorporates the task background, data collection, model details, evaluation metrics, and failure case analysis. Figure S1: the accuracy concerning the number of training epochs for the “black-box” model and deep keypoint stadiometry model. Figure S2: comparison of the occluder size prediction with MAE (the smaller, the better) and QWK (the larger, the better) metrics. Supplementary Table 1: the statistics of the clinical characteristics of collected ASD patients. [file 9790653.f1.zip › Research(supplymentary)/images/lancetsample3333.png]

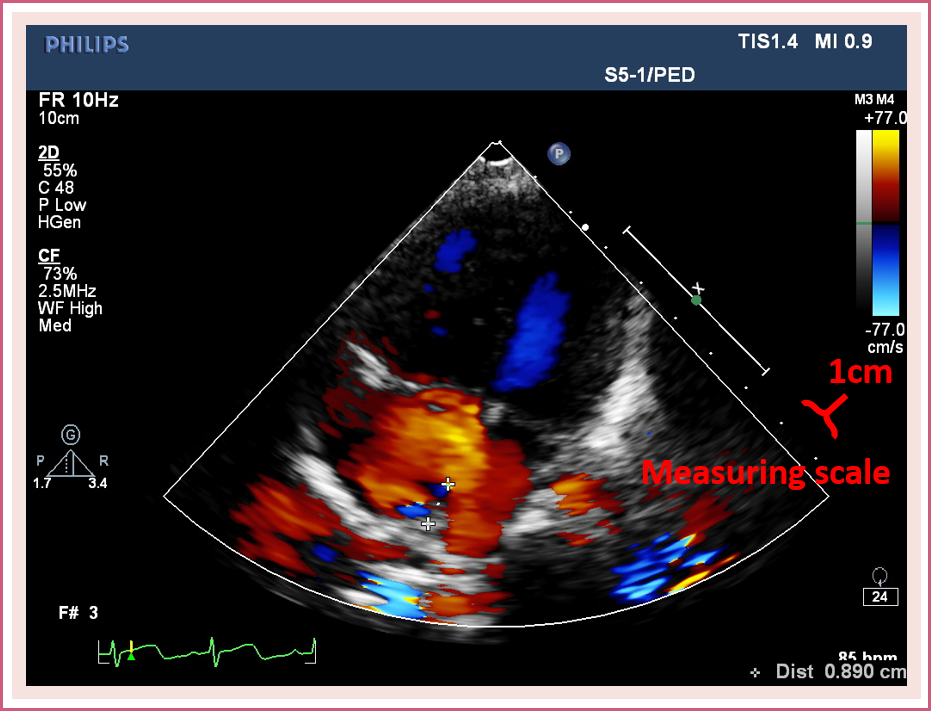

Supplement: Supplementary Materials — The supplementary incorporates the task background, data collection, model details, evaluation metrics, and failure case analysis. Figure S1: the accuracy concerning the number of training epochs for the “black-box” model and deep keypoint stadiometry model. Figure S2: comparison of the occluder size prediction with MAE (the smaller, the better) and QWK (the larger, the better) metrics. Supplementary Table 1: the statistics of the clinical characteristics of collected ASD patients. [file 9790653.f1.zip › Research(supplymentary)/images/lancetscale.png]

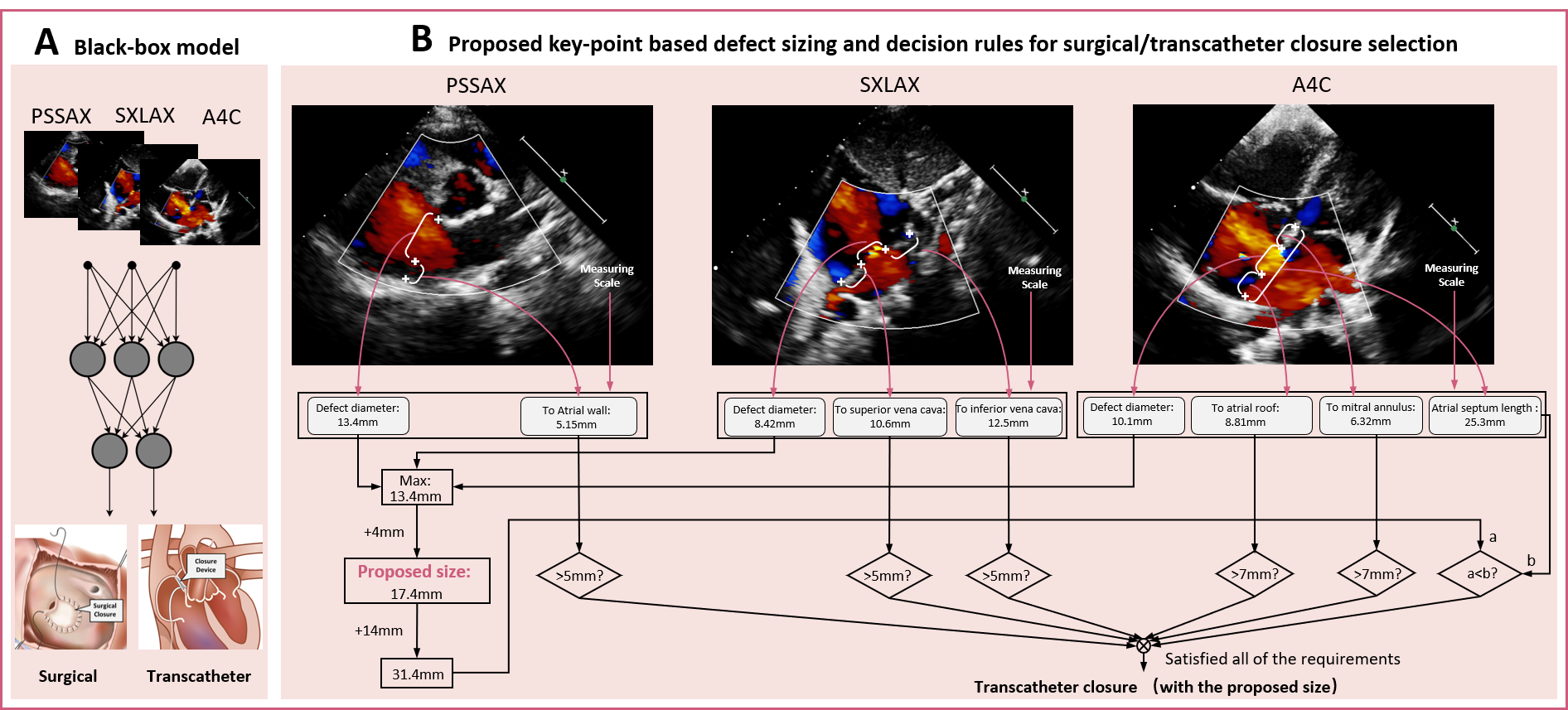

Supplement: Supplementary Materials — The supplementary incorporates the task background, data collection, model details, evaluation metrics, and failure case analysis. Figure S1: the accuracy concerning the number of training epochs for the “black-box” model and deep keypoint stadiometry model. Figure S2: comparison of the occluder size prediction with MAE (the smaller, the better) and QWK (the larger, the better) metrics. Supplementary Table 1: the statistics of the clinical characteristics of collected ASD patients. [file 9790653.f1.zip › Research(supplymentary)/images/Picture3.png]

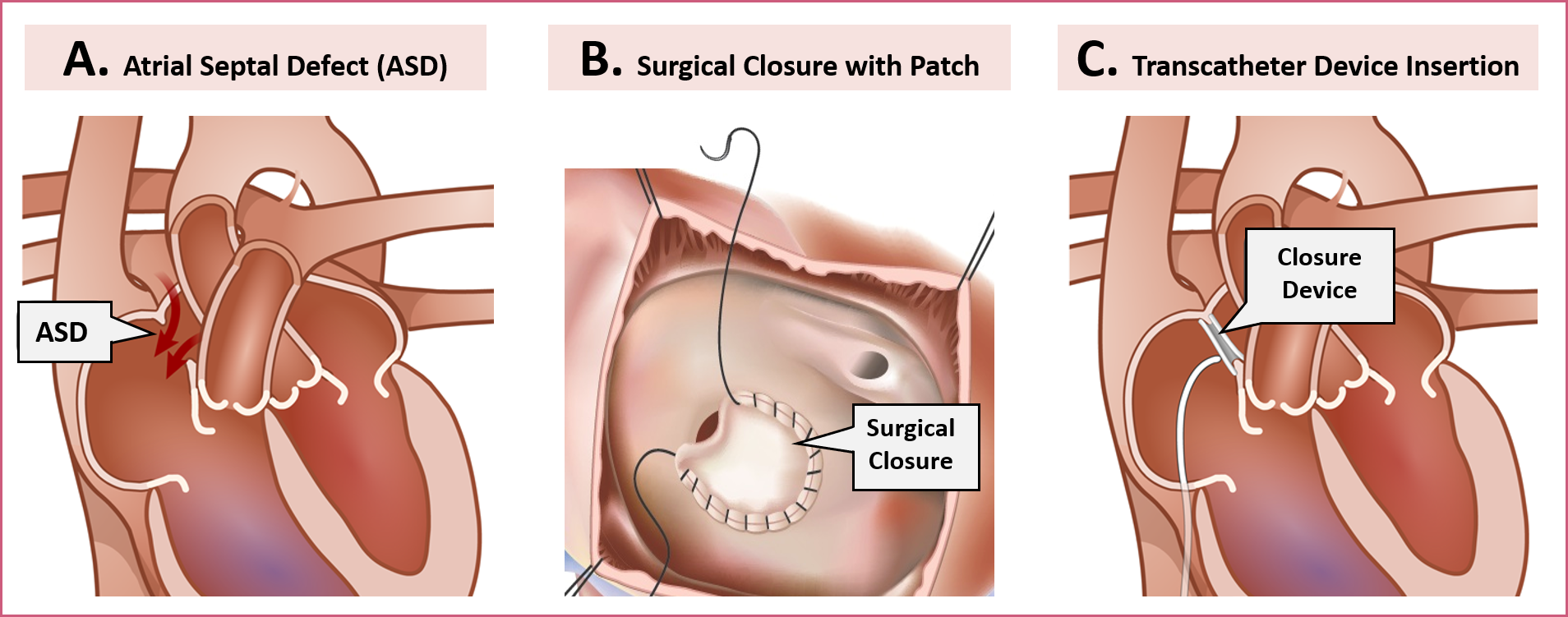

Supplement: Supplementary Materials — The supplementary incorporates the task background, data collection, model details, evaluation metrics, and failure case analysis. Figure S1: the accuracy concerning the number of training epochs for the “black-box” model and deep keypoint stadiometry model. Figure S2: comparison of the occluder size prediction with MAE (the smaller, the better) and QWK (the larger, the better) metrics. Supplementary Table 1: the statistics of the clinical characteristics of collected ASD patients. [file 9790653.f1.zip › Research(supplymentary)/images/Picture4.png]

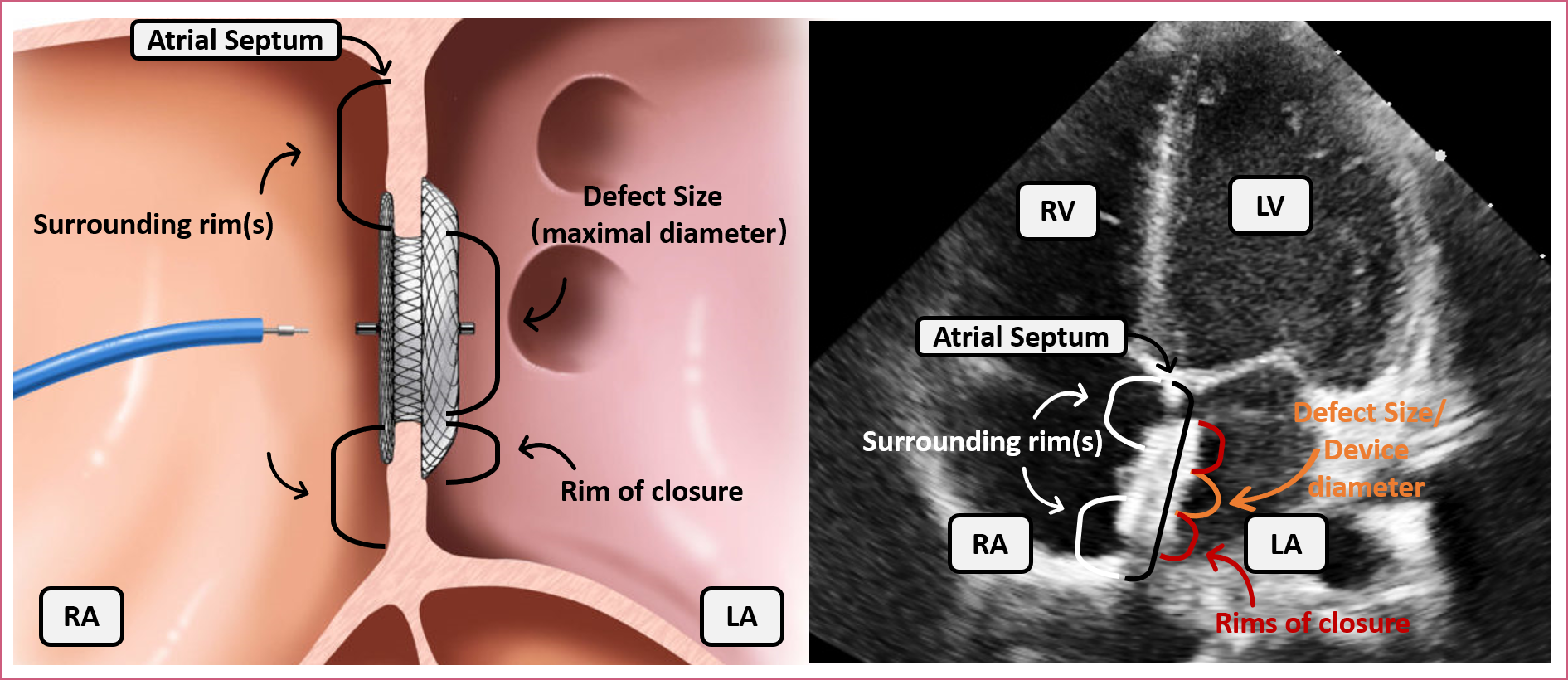

Supplement: Supplementary Materials — The supplementary incorporates the task background, data collection, model details, evaluation metrics, and failure case analysis. Figure S1: the accuracy concerning the number of training epochs for the “black-box” model and deep keypoint stadiometry model. Figure S2: comparison of the occluder size prediction with MAE (the smaller, the better) and QWK (the larger, the better) metrics. Supplementary Table 1: the statistics of the clinical characteristics of collected ASD patients. [file 9790653.f1.zip › Research(supplymentary)/images/Picture5.png]

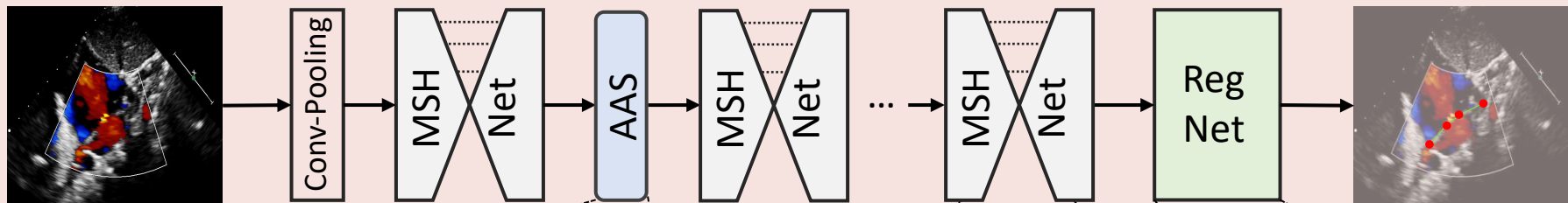

### Anatomical-aware Supervisions

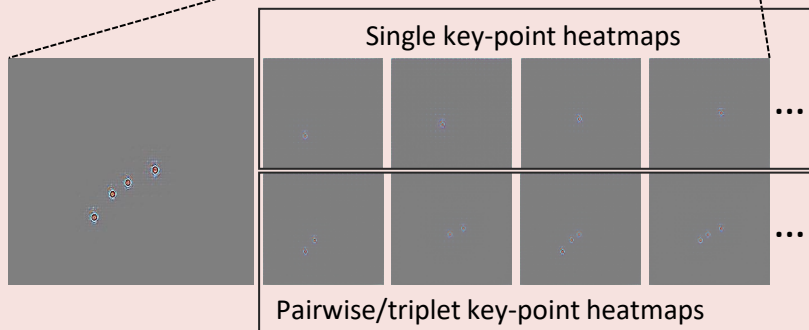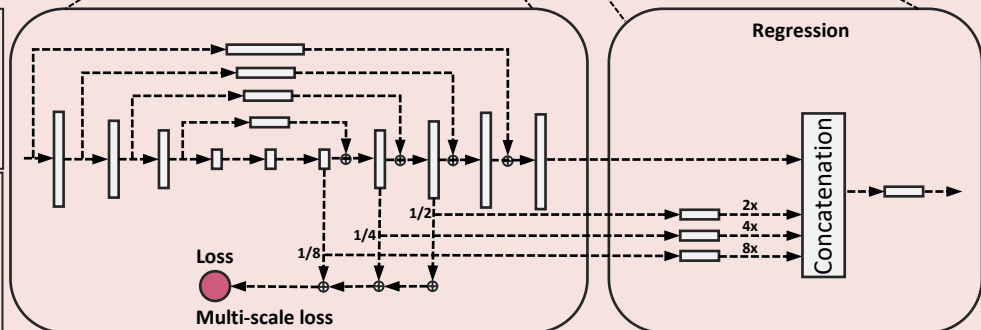

Supplement: Supplementary Materials — The supplementary incorporates the task background, data collection, model details, evaluation metrics, and failure case analysis. Figure S1: the accuracy concerning the number of training epochs for the “black-box” model and deep keypoint stadiometry model. Figure S2: comparison of the occluder size prediction with MAE (the smaller, the better) and QWK (the larger, the better) metrics. Supplementary Table 1: the statistics of the clinical characteristics of collected ASD patients. [file 9790653.f1.zip › Research(supplymentary)/images/picture6.pdf]

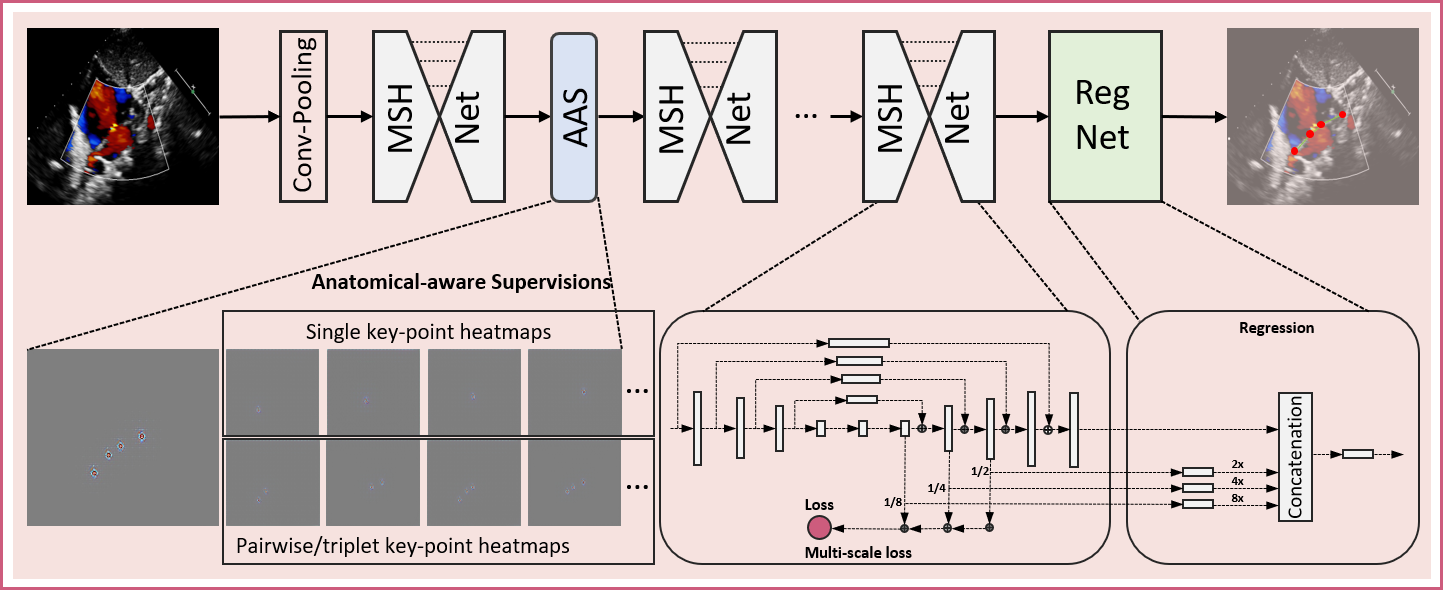

Supplement: Supplementary Materials — The supplementary incorporates the task background, data collection, model details, evaluation metrics, and failure case analysis. Figure S1: the accuracy concerning the number of training epochs for the “black-box” model and deep keypoint stadiometry model. Figure S2: comparison of the occluder size prediction with MAE (the smaller, the better) and QWK (the larger, the better) metrics. Supplementary Table 1: the statistics of the clinical characteristics of collected ASD patients. [file 9790653.f1.zip › Research(supplymentary)/images/Picture6.png]

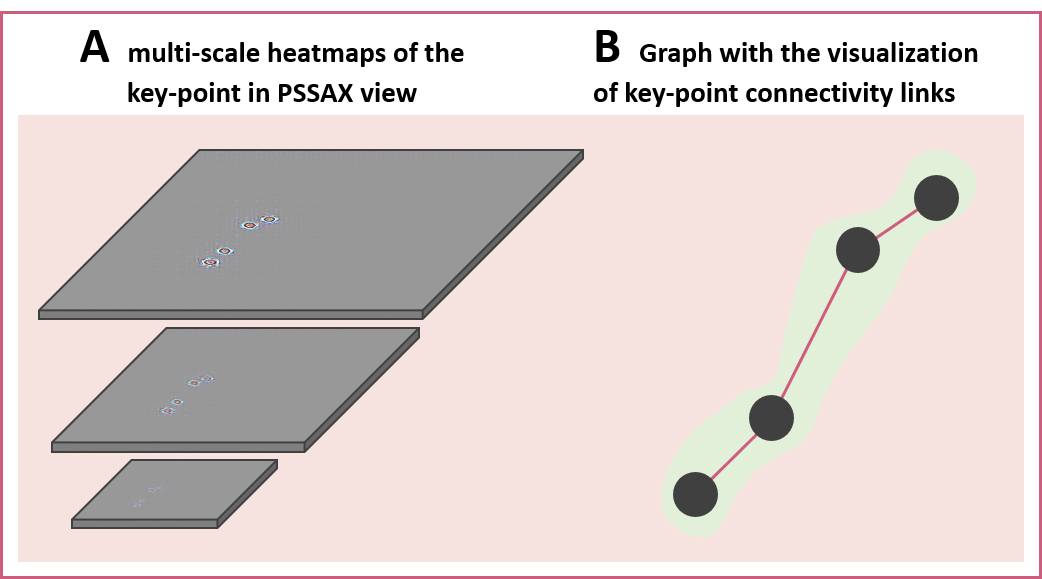

Supplement: Supplementary Materials — The supplementary incorporates the task background, data collection, model details, evaluation metrics, and failure case analysis. Figure S1: the accuracy concerning the number of training epochs for the “black-box” model and deep keypoint stadiometry model. Figure S2: comparison of the occluder size prediction with MAE (the smaller, the better) and QWK (the larger, the better) metrics. Supplementary Table 1: the statistics of the clinical characteristics of collected ASD patients. [file 9790653.f1.zip › Research(supplymentary)/images/Picture66.png]

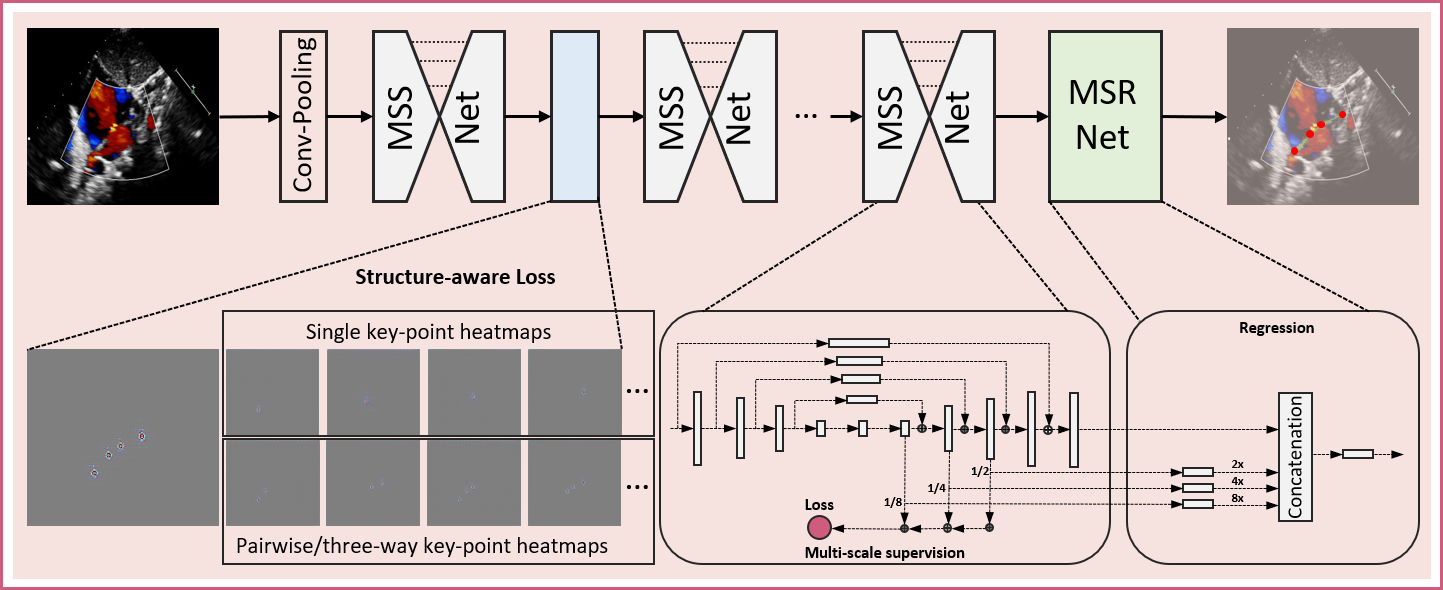

Supplement: Supplementary Materials — The supplementary incorporates the task background, data collection, model details, evaluation metrics, and failure case analysis. Figure S1: the accuracy concerning the number of training epochs for the “black-box” model and deep keypoint stadiometry model. Figure S2: comparison of the occluder size prediction with MAE (the smaller, the better) and QWK (the larger, the better) metrics. Supplementary Table 1: the statistics of the clinical characteristics of collected ASD patients. [file 9790653.f1.zip › Research(supplymentary)/images/Picture7.png]
